# Imputation of missing values for electronic health record laboratory data

**DOI:** 10.1038/s41746-021-00518-0

**Published:** 2021-10-11

**Authors:** Jiang Li, Xiaowei S. Yan, Durgesh Chaudhary, Venkatesh Avula, Satish Mudiganti, Hannah Husby, Shima Shahjouei, Ardavan Afshar, Walter F. Stewart, Mohammed Yeasin, Ramin Zand, Vida Abedi

**Affiliations:** 1 Geisinger Health System, Danville, PA USA; 2 Sutter Center for Health System Research, Walnut Creek, CA USA; 3 School of Computing, Georgia Institute of Technology, Atlanta, GA USA; 4 Medcurio, 300 Frank H Ogawa Plaza, Suite 248, Oakland, CA 94612 USA; 5 Department of Electrical & Computer Engineering, University of Memphis, Memphis, TN USA; 6 Department of Public Health Sciences, College of Medicine, The Pennsylvania State University, Hershey, PA USA

**Keywords:** Laboratory techniques and procedures, Data processing, Cardiovascular diseases, Machine learning

## Abstract

Laboratory data from Electronic Health Records (EHR) are often used in prediction models where estimation bias and model performance from missingness can be mitigated using imputation methods. We demonstrate the utility of imputation in two real-world EHR-derived cohorts of ischemic stroke from Geisinger and of heart failure from Sutter Health to: (1) characterize the patterns of missingness in laboratory variables; (2) simulate two missing mechanisms, arbitrary and monotone; (3) compare cross-sectional and multi-level multivariate missing imputation algorithms applied to laboratory data; (4) assess whether incorporation of latent information, derived from comorbidity data, can improve the performance of the algorithms. The latter was based on a case study of hemoglobin A1c under a univariate missing imputation framework. Overall, the pattern of missingness in EHR laboratory variables was *not at random* and was highly associated with patients’ comorbidity data; and the multi-level imputation algorithm showed smaller imputation error than the cross-sectional method.

## Introduction

Laboratory data are often used in machine-learning-enabled EHR-based clinical decision support systems^1–4^ and significantly improve disease modeling and outcome prediction^3,5–8^. However, laboratory data are often missing for intentional (e.g., the patient does not need certain laboratory tests) or unintentional (e.g., lack of routine checkup or follow-up) reasons, and this missingness can result in loss of power, biased estimates^9,10^, and models that underperform. Notably, imputing missing values for EHR laboratory variables, which includes irregular time-series data, is a persistent challenge. Missing patterns and missingness mechanisms for laboratory data have not been well characterized. Moreover, the imputation strategy that is optimal given a defined missingness pattern has not been studied.

Within clinical trial frameworks or observational studies, various imputation models have been successfully applied and these include mean substitution, regression, hot deck^11^, tree-based^12^, as well as advanced statistical methods, such as expectation maximization (EM)^13^, full information maximum likelihood (FIML)^14^, and multiple imputations (MI)^15,16^. In general, imputation algorithms that rely on inter-attribute correlations perform better. The data correlation could exist within a time point across all samples (cross-sectional) or between time points at an individual level (longitudinal), within a single variable (univariate) or between variables (multivariate), and missing in one variable correlated to observation in other variables and vice versa. MI, the commonly used imputation method, assumes that each missing value has a distribution of plausible values, which reflect the uncertainty of the missing value. MI is usually conducted using three procedures, fully conditional specification (FCS)^17–19^, joint model (JM)^20^, and monotone imputation^21^. Multivariate Imputation by Chained Equations (MICE)^22^—a widely used open-source imputation software with built-in cross-sectional and multi-level univariate or multivariate algorithms – is applied to laboratory variables from EHR. Previous studies applying MICE or other methods to impute one laboratory variable with common laboratory variables in cross-sectional studies have achieved some promising results^23–26^.

**Fig. 1:**
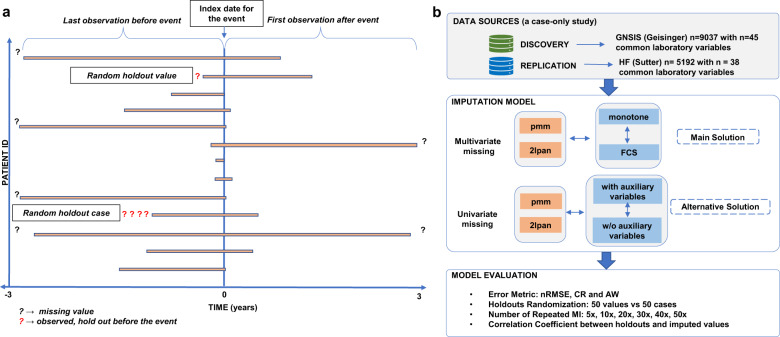
Data extraction and analysis pipeline. **1a** Inspired by a stepwise imputation by observation blocks in longitudinal data from EHR, we extracted the last observation before an event (e.g., stroke or heart failure), and the first observation after the event. Two types of holdouts, random holdout values (HV) and random holdout complete cases (HC), represent two missingness scenarios, MAR and monotone with NMAR, respectively. **1b** Outline the tested imputation models and algorithms evaluated by error metrics and the results after repeated multiple imputation (MI). Abbreviations: PMM predictive mean matching, 2lpan Implements the Gibbs sampler for the linear two-level model with homogeneous within-group (patient ID) variances, LMM linear mixed-effects model, FCS fully conditional specification, nRMSE Root Mean Square Error normalized by standard deviation, CR Coverage rate, the proportion of confidence intervals that contain the true value.

**Fig. 2:**
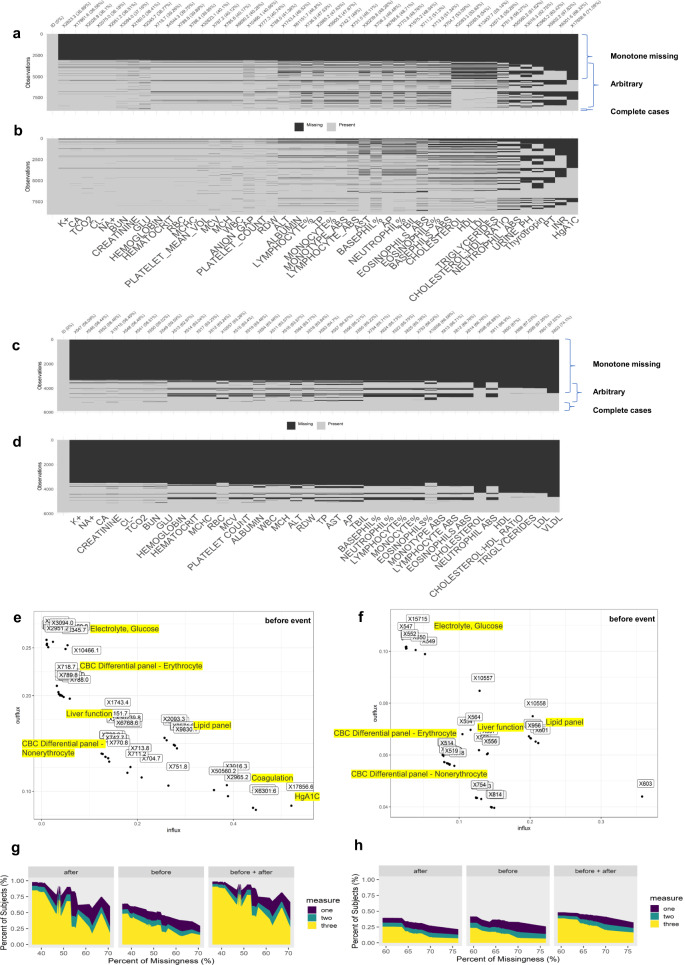
Exploring missingness pattern and mechanism: level of missingness in GNSIS (n=9037) and HF (=5192). **a**, **b**/**c**, **d** Missingness pattern, monotone with some degree of randomness, before or after the event was created by R “naniar” package (black represents missing and light gray represents observed). The *x*-axis is the description of the laboratory variables, which are sorted based on the percentage of missingness. The corresponding laboratory component ID and % missing was labeled on the top row. Some patients were marked as both monotone and arbitrary missing because some providers ordered a specific laboratory test (or a specific panel of tests) for some patients for a variety of reasons under certain circumstances. This would violate the sequentially ordering of laboratory tests and lead to some random missing (arbitrary) for other patients. **e**, **f** A fluxplot showing the distribution of laboratory variables before the event is determined by the corresponding influx and outflux values. The laboratory variables in a panel test generally were clustered together. **g**, **h** Area plot (identity but not stacking) to show the percent of subjects with repeated (up to three) measures. The *x*-axis was laboratory variables sorted by percent of missingness (%) before the event.

The key questions when deciding on imputation techniques for laboratory variables are the following. (1) What is the pattern or mechanism of missingness in these variables; (2) How to choose the algorithms and procedures for imputation of missingness; (3) How well to impute laboratory data in a cross-sectional design compared to a longitudinal design; (4) Can auxiliary variables, based on comorbidity information, be useful in the imputation model; and (5) How well the conclusion made from a single dataset is applied to an independent dataset with different setup or missingness pattern—namely generalizability. In this study, we determine patterns and explore mechanisms of missingness in laboratory variables in Geisinger Healthcare System in Pennsylvania, and Sutter Health in California (Fig. 1) for two distinct cases. We evaluate the performance of commonly used imputation algorithms with a focus on model-based MI frameworks that could accommodate high missingness rates (>50%). We simulate two mechanisms of missingness, arbitrary and monotone, by randomly holding-out laboratory values (HV) and complete patient records (HC), to mimic different patterns of missingness observed in EHRs (Fig. 2a–d), and evaluate the performance of the algorithms. Finally, we use a case study to assess the value of applying latent information derived from comorbidity as auxiliary variables to predict hemoglobin A1c (HbA1c).

## Result

### Laboratory measures characteristics

Overall, 45 quantitative laboratory variables from GNSIS (*n* = 9037) and 38 from HF (*n* = 5192) with <75% missingness were analyzed in this study. Kernel density plot was used to illustrate the data distribution for each variable before the index date (Supplementary Fig. 1). The laboratory variables from two EHR datasets were summarized in Table 1 (See supplementary Table 1 for detailed information).

**Table 1:** Summary of laboratory variables with missingness <75% before the event from two EHR datasets.

LONIC ID	Component	Ischemic stroke (Geisinger, *n* = 9037)					Heart Failure (Sutter Health, *n* = 5192)				
		Missing% before event^a^	Missing% after event	Mean (SD) before event	Mean (SD) after event	Corr	Missing% before event	Missing% after event	Mean (SD) before event	Mean (SD) after event	Corr
*1742-6*	Alanine aminotransferase						63.77	67.69	32.903 (13.526)	30.757 (13.142)	0.412
*1743-4*	Alanine aminotransferase	46.52	25.10	21.683 (10.840)	22.661 (12.121)	0.453					
*1751-7*	Albumin						63.46	66.76	3.603 (0.435)	3.611 (0.387)	0.495
*61151-7*	Albumin	46.80	20.49	3.941 (0.501)	3.750 (0.517)	0.535					
*6768-6*	Alkaline phosphatase	48.71	24.42	81.756 (27.609)	78.844 (27.251)	0.672	65.21	68.47	90.617 (29.471)	91.361 (32.130)	0.631
*10466-1*	Anion gap 3	40.66	4.26	10.515 (3.218)	10.17 (3.170)	0.443					
*1920-8*	Aspartate aminotransferase						64.87	68.22	23.651 (9.351)	23.399 (8.971)	0.430
*30239-8*	Aspartate aminotransferase	48.28	26.20	24.519 (8.921)	25.530 (9.762)	0.385					
*704-7*^*b*^	Basophils	53.09	16.73	−1.452 (0.296)	−1.497 (0.292)	0.517					
*706-2*^*b*^	Basophils/100 leukocytes	48.48	9.90	−7.679 (10.008)	−8.652 (10.205)	0.402	65.71	68.71	0.486 (0.553)	0.492 (0.556)	0.398
*1975-2*	Bilirubin	48.94	25.06	0.531 (0.291)	0.577 (0.314)	0.550	65.22	68.89	0.554 (0.261)	0.571 (0.259)	0.566
*17861-6*	Calcium	36.08	2.26	9.272 (0.55)	8.924 (0.522)	0.416	58.46	60.52	9.004 (0.481)	9.031 (0.466)	0.448
*2028-9*	Carbon dioxide	36.10	2.14	26.834 (3.035)	25.446 (3.121)	0.467	58.51	60.49	27.806 (3.038)	28.081 (3.177)	0.494
*2075-0*	Chloride	36.18	2.25	101.975 (3.925)	103.781 (4.128)	0.507	58.49	60.49	103.437 (3.727)	103.003 (3.982)	0.514
*2093-3*	Cholesterol	53.62	16.60	178.579 (46.356)	166.091 (45.681)	0.597	66.88	72.73	164.172 (42.202)	159.966 (42.207)	0.590
*2085-9*	Cholesterol in HDL	54.00	16.76	48.050 (14.786)	44.925 (14.348)	0.720	67.00	72.72	51.009 (16.625)	50.845 (16.035)	0.764
*13457-7*	Cholesterol in LDL	55.14	18.46	99.853 (38.611)	92.945 (38.177)	0.586	67.52	73.11	87.573 (34.429)	83.182 (34.584)	0.569
*9830-1*	Cholesterol.total/Cholesterol.in HDL	55.47	19.43	3.958 (1.393)	3.948 (1.433)	0.683	67.03	72.87	3.444 (1.173)	3.342 (1.116)	0.631
*5902-2*	Coagulation tissue factor induced	67.83	29.91	14.233 (1.839)	14.379 (1.642)	0.456					
*6301-6*	Coagulation tissue factor induced.INR	68.32	30.59	1.108 (0.173)	1.124 (0.161)	0.450					
*2160-0*	Creatinine	38.41	6.16	1.009 (0.325)	0.954 (0.321)	0.804	58.49	61.06	1.06 (0.369)	1.147 (0.434)	0.723
*711-2*^*b*^	Eosinophils	51.30	15.77	−0.828 (0.388)	−0.918 (0.426)	0.381	66.76	68.98	0.209 (0.145)	0.210 (0.149)	0.506
*713-8*^*b*^	Eosinophils/100 leukocytes	51.34	20.02	0.325 (0.347)	0.254 (0.385)	0.353	66.02	69.11	2.886 (1.927)	2.947 (2.026)	0.454
*788-0*	Erythrocyte distribution width	41.36	4.43	13.944 (1.307)	13.837 (1.246)	0.770	63.84	66.19	14.292 (1.332)	14.526 (1.486)	0.571
*785-6*	Erythrocyte mean corpuscular hemoglobin	40.17	3.18	30.294 (2.113)	30.323 (2.084)	0.876	63.57	65.95	29.946 (2.129)	29.856 (2.323)	0.778
*786-4*	Erythrocyte mean corpuscular hemoglobin concentration	39.95	2.80	33.425 (1.239)	33.597 (1.190)	0.699	63.23	65.78	32.648 (1.233)	32.468 (1.258)	0.610
*787-2*	Erythrocyte mean corpuscular volume	40.12	3.07	90.546 (5.439)	90.162 (5.324)	0.861	63.40	65.85	91.673 (5.859)	91.888 (6.060)	0.780
*789-8*	Erythrocytes	39.89	2.79	4.329 (0.637)	4.289 (0.610)	0.731	63.24	65.80	4.304 (0.587)	4.24 (0.575)	0.664
*2345-7*	Glucose	38.77	4.32	119.727 (38.239)	120.475 (36.528)	0.452	59.59	61.89	113.58 (33.589)	113.365 (32.696)	0.413
*4544-3*	Hematocrit	39.75	2.68	39.048 (5.377)	38.556 (5.178)	0.698	63.04	65.66	39.292 (5.078)	38.786 (4.992)	0.617
*718-7*	Hemoglobin	39.26	2.61	13.081 (1.943)	12.965 (1.891)	0.728	62.97	65.63	12.836 (1.821)	12.614 (1.804)	0.637
*17856-6*	Hemoglobin A1c/Hemoglobin.total	71.09	40.69	7.025 (1.633)	6.489 (1.502)	0.743					
*6690-2*	Leukocytes	40.28	4.11	8.079 (2.665)	8.559 (2.939)	0.460	63.57	66.10	7.475 (2.424)	7.341 (2.306)	0.518
*731-0*	Lymphocytes	48.11	9.25	1.726 (0.777)	1.732 (0.778)	0.628	66.76	68.88	1.698 (0.666)	1.624 (0.670)	0.633
*736-9*	Lymphocytes/100 leukocytes	47.53	8.75	22.22 (9.565)	21.644 (10.113)	0.503	65.75	68.71	23.995 (9.045)	23.283 (9.288)	0.518
*742-7*	Monocytes	48.00	9.45	0.681 (0.276)	0.71 (0.289)	0.451	66.71	68.88	0.672 (0.253)	0.662 (0.245)	0.513
*5905-5*	Monocytes/100 leukocytes	47.67	9.11	8.486 (2.844)	8.43 (2.756)	0.413	65.78	68.76	9.19 (2.693)	9.249 (2.840)	0.487
*751-8*	Neutrophils	56.27	38.43	5.451 (2.424)	5.866 (2.736)	0.372	66.90	69.08	4.783 (1.992)	4.725 (1.897)	0.401
*770-8*	Neutrophils/100 leukocytes	48.76	10.05	65.687 (11.447)	66.926 (12.089)	0.412	65.73	68.69	63.234 (10.551)	63.738 (10.894)	0.436
*32623-1*	Platelet mean volume	40.10	2.85	9.854 (1.397)	9.746 (1.328)	0.752					
*777-3*	Platelets	40.12	3.07	232.984 (75.418)	216.817 (68.570)	0.723	63.46	66.09	228.131 (70.929)	226.625 (74.628)	0.660
*2823-3*	Potassium	35.89	2.40	4.256 (0.481)	4.008 (0.447)	0.328	58.09	60.39	4.262 (0.463)	4.281 (0.484)	0.391
*2885-2*	Protein	47.63	22.66	6.874 (0.647)	6.659 (0.673)	0.448	64.70	68.15	7.153 (0.585)	7.173 (0.584)	0.525
*2951-2*	Sodium	36.51	2.37	139.234 (3.318)	139.361 (3.181)	0.461	58.44	60.42	139.45 (3.184)	139.271 (3.384)	0.473
*3016-3*	Thyrotropin	62.75	44.23	2.249 (1.485)	2.311 (1.600)	0.478					
*2571-8*	Triglyceride	55.28	17.90	142.896 (70.842)	131.657 (67.640)	0.603	67.35	73.22	120.268 (61.561)	122.985 (64.578)	0.581
*3094-0*	Urea nitrogen	37.16	4.02	19.239 (8.073)	17.617 (7.760)	0.622	59.02	61.13	21.187 (9.096)	23.187 (10.671)	0.599
*50560-2*	Urine pH	61.62	36.73	6.009 (0.790)	6.034 (0.773)	0.189					
*13458-5*	Cholesterol in VLDL						74.10	78.43	24.308 (12.754)	25.067 (13.391)	0.610

*LONIC ID* Logical Observation Identifiers Names and Codes, *Corr* the correlation coefficient of laboratory variables between before and after event. *SD* standard deviation.

^a^Event represents either ischemic stroke or Heart Failure in the corresponding datasets.

^b^The log transformation has been conducted in the following laboratory variables (704-7, 706-2, 713-8, and 711-2) in Ischemic stroke dataset from Geisinger due to the original exponential distribution of these laboratory variables.

For variables collected as a panel (e.g., CBC, electrolyte, liver function, kidney function, lipid panel, and metabolic panel), their missingness usually occurred concurrently (Fig. 2e, f). The selection of laboratory variables for imputation was determined by the correlation matrix and the connection between missingness and observation among the variables visualized by a fluxplot. The pairwise correlation between two observations (before or after the index date) was moderate (|R | ≈ 0.5) across all variables (Supplementary Table 1). On the other hand, there were low correlation coefficients (|R | < 0.2) between selected variables from each test panel, however, this correlation was still statistically significant (Supplementary Fig. 6). According to the fluxplot (Fig. 2e, f), electrolyte and glucose levels had the highest *O*_*jk*_, suggesting their observed data connected to the missing data of other variables, whereas HbA1c and coagulation related variables have a highest *I*_*jk*_, suggesting their missingness was connected to the observed data from other variables. All these laboratory variables were included in the MI procedure.

### Analyses of missingness patterns and mechanisms

Missingness before (Fig. 2a, c) or after (Fig. 2b, d) the index date, was likely to be “monotone” with some degree of randomness. As summarized in Fig. 2, we noticed that (1) the missingness was higher before the index date than after in the GNSIS dataset, (2) the HF data had a higher percent of missingness for both before and after the index date compared to the GNSIS dataset, (3) only a small portion of patients have repeated measurements (see Fig. 2g, h for the percentage of subjects with greater than one measurement), and (4) the missingness level was reduced by combining data from before and after only in the GNSIS dataset (Fig. 2g, h).

Further analysis of the pattern of missingness was performed using margin plots. We assessed the missingness pattern between “before the index date” and “after the index date” or between two different laboratory variables (Supplementary Fig. 2). We randomly selected four laboratory variables, one from each panel with a different level of missingness. The pattern of laboratory measures did not violate MAR. Under the MAR assumption, the distributions showed in the side boxplot of one laboratory variable, conditioned on the status of observed (blue) or missing in the other laboratory variable, could be different, both in location (median) and spread (IQR). However, clusters were not formed in the scatterplots, and no significant shift in the boxplot between missing (red) and observed (blue) values were detected. (Supplementary Fig. 2).

**Fig. 3:**
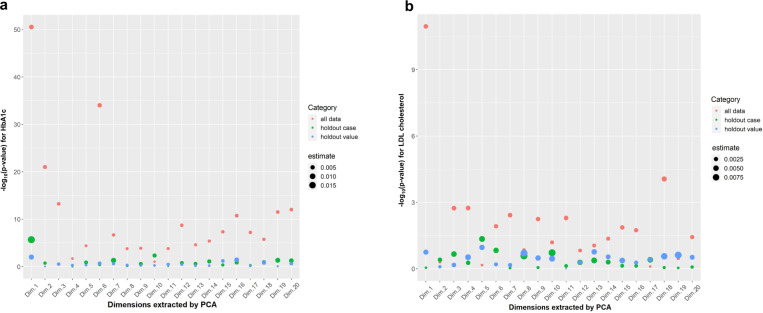
This missingness (dummy variables) was highly associated with comorbidity. HbA1C (**a**) in GNSIS dataset and LDL cholesterol (**b**) in HF dataset was one of the most valuable laboratory variables used in the prediction model for the outcome of interest in ischemic stroke and/or heart failure. PCA was conducted by R “factoextra“ package. Dim represented the dimensions extracted from the comorbidity matrix using prcomp function; The *y*-axis represented log-transformed *p*-value of the significance of the difference (absolute value) in principal component values between observed and missing groups after Welch unpaired *t*-test.

The co-analysis of patient comorbidities and missingness of laboratory measurements revealed that the missingness was related to disease burden, and the patients with higher disease burden had less missingness in both the GNSIS and HF datasets. For each laboratory variable, the association between missingness and each main PC (labeled as Dim) was extracted from the comorbidity matrix (Fig. 3a, b). Patients with observed laboratory values had significantly higher PC values (red dots) than patients with a missing value.

We studied two simulation policies, by holding-out 50 random laboratory values (HV) and 50 complete patient records (HC), to mimic different patterns of missingness. The GNSIS dataset included 393 completed cases, while the HF included 777 complete cases from which 50 HC were randomly drawn for each cohort. When analyzing the 50 HV per variable our results showed no significant association with any of the PCs across all the variables (blue dots), suggesting MAR pattern; however, analysis of the 50 HC showed a significantly higher PC value (green dots) at least for the first main PC (labeled as Dim.1) in the GNSIS (Fig. 3a and Supplementary Fig. 7a) but not in the HF dataset (Fig. 3b and Supplementary Fig. 7b). These observations highlight the fact that patterns of missingness can have unique attributes based on the originating centers and associated phenotypes.

### Coverage rate comparison among different imputation models

**Fig. 4:**
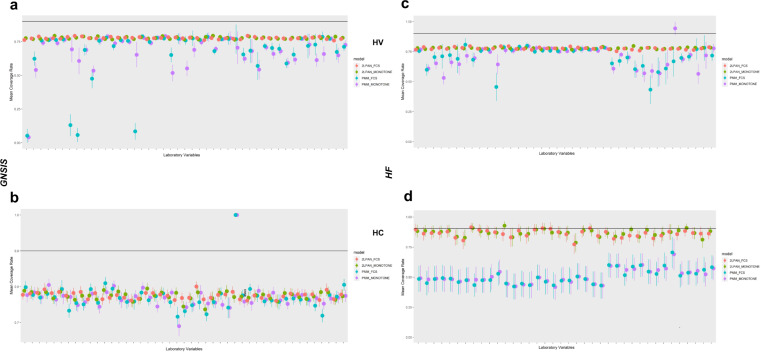
Mean coverage rate (CR) across all imputation algorithms evaluated for GNSIS and HF datasets. The vertical bar represents 95%CI of the corresponding mean imputed value for 50 holdouts. The horizontal line represents the mean coverage rate of 0.90. **a**/**b** represents CR for 50 holdout values (HV) or complete cases (HC), respectively, in GNSIS; **c**/**d** represents CR for 50 HV or HC, respectively, in HF.

The variability (95%CI) of the mean coverage rate (CR) was generally higher for PMM than 2l.pan algorithms. The mean CR for 50 holdouts representing the proportion of confidence intervals (CI) that contain the true value in the two simulation policies was evaluated for both datasets and all the imputation algorithms included in this study (Fig. 4). For both policies (HV and HC), the 2l.pan-FCS and 2l.pan-monotone showed better CR than cross-sectional PMM-FCS and PMM-monotone imputation (see Fig. 4a for GNSIS and Fig. 4b for HF). Finally, the results obtained from the average width were consistent with CR (Supplementary Fig. 8).

### Uncertainty propagation

**Fig. 5:**
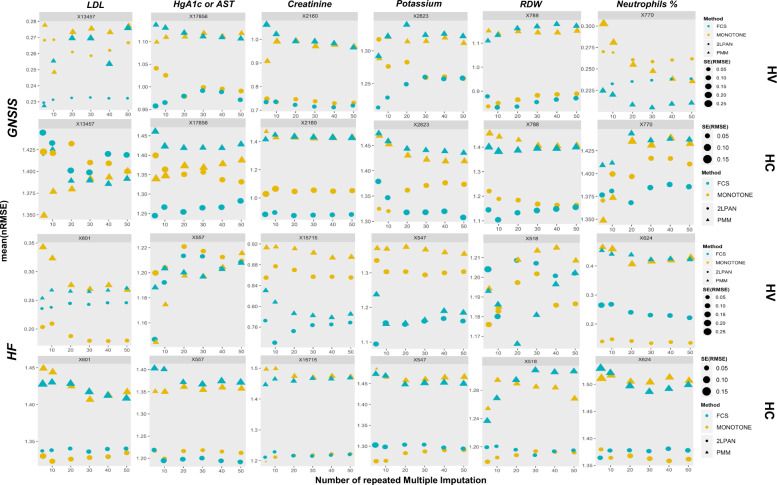
Laboratory test, missingness pattern, and imputation algorithm dependent uncertainty propagation during 50 repeated multiple imputation (MI) for holdouts using RMSE. The *y*-axis was labeled with mean(nRMSE) after the number of MI. This mean of normalized RMSE in *y*-axis represents the error over the number of runs. The size of the triangle or round dot represents the standard error of RMSE over the number of runs. “L” shaped or inverse “L” shaped distribution of the mean(nRMSE) after 50 runs of MI suggested this uncertainty reached a plateau. However, for some variables (e.g., 13457-7 of LDL or 17856-6 of HgA1C) in HC, the uncertainty went upward but not reached a statistical convergence.

The nRMSE after repeated MI was dynamically assessed to determine the level and speed of the uncertainty that was propagated after 5, 10, 20, 30, 40, 50 repeated imputation. Our results (Fig. 5) showed that (1) the mean and standard error of nRMSE for HC were generally larger when compared to HV; (2) for HV, mean nRMSE stabilized after 30 repeats for most of the variables, and the standard error of nRMSE stabilized after 20 or 30 repeats as well; (3) in general, FCS performed better than monotone, and 2l.pan performed better than PMM for the majority of variables in both datasets; and (4) for HC, mean nRMSE did not converge for some of the variables even with 50 runs. This latter observation highlights that when missing follows an MNAR pattern a higher number of runs are needed to ensure that the nRMSE are stabilized.

### Performance evaluation of model

**Fig. 6:**
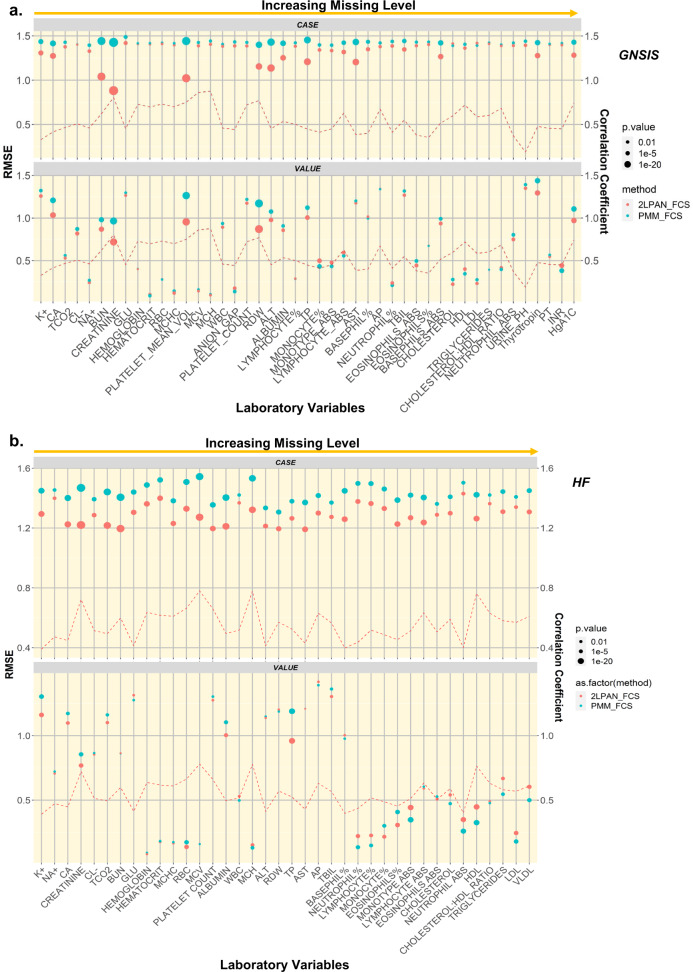
Comparing the performance of selected imputation algorithms after 50 repeated multiple imputation for random holdout cases (HC) or values (HV) using nRMSE in GNSIS and HF datasets. Levene’s test showed an equal variance of the nRMSE from two compared imputation algorithms, e.g. 2LPAN-FCS and PMM-FCS. Shapiro-Wilk test showed the normality of the difference for each comparison. An unpaired *t*-test was conducted to determine the mean difference of nRMSE between two compared imputation algorithms. Only the raw *p*-value < 0.05/45 (~0.0011) for GNSIS (**a**) or < 0.05/38 (~0.0013) for HF (**b**) was considered as statistical significance, which survived the Bonferroni correction for multiple testing. The curve for Pearson’s correlation coefficient of observed values between before and after index date per variable was overlaid to the corresponding dot plot for normalized RMSE.

We assessed the model performance for different algorithms and simulation policies. Overall, for the HV simulation policy, FCS performed better than monotone in both datasets (Supplementary Fig. 5a, b). However, the improved performance of FCS over the monotone procedure was unclear for the HC simulation policy, particularly in the HF dataset. The multi-level (2l.pan) imputation outperformed the cross-sectional PMM, as indicated by a significantly lower nRMSE (after correction for multiple testing) in Fig. 6. Given 50 HVs in GNSIS, we observed that 21 out of the 45 (46.7%) variables showed a significantly lower nRMSE for 2l.pan-FCS than that for 2l.pan-monotone. This number was 8 out of 45 (17.8%) for the 50 HCs. Similarly, we identified 10 out of 45 (22.2%) variables having significantly lower nRMSE for 2l.pan-FCS than that for PMM-FCS, while 15 out of 45 (33.3%) variables for 50 HCs showed similar results. Analysis of the HF dataset corroborated similar observations; in particular, 17 out of the 38 (44.7%) variables showed a significantly lower nRMSE for 2l.pan-FCS than that for 2l.pan-monotone. We also identified 8 out of the 38 variables having significantly lower nRMSE for 2l.pan-FCS than that for PMM-FCS. The laboratory variables in the same panel (e.g., electrolyte, lipid panel, CBC) showed similar patterns (Fig. 6).

Finally, our comprehensive analysis, including uncertainty assessment, showed that the standard error of imputed values and their deviation from the regression line, estimated by the correlation coefficient (*R*), was higher in HC simulation policy across all laboratory variables for both datasets. The latter was shown by the over-imputation plots (Supplementary Fig. 4). This observation emphasizes the need for a more careful assessment of uncertainty when analyzing laboratory variables with MNAR patterns.

### A case study for hemoglobin A1c

We designed a case study to assess the practical value of improvement in imputation for the laboratory measurement of Hemoglobin A1c (HbA1c, LOINC ID: 17856-6), which had the highest missingness level. HbA1c has also the highest *I*_*jk*_, suggesting its missing data connects to the observed data from other variables in a multivariate MI model.

**Fig. 7:**
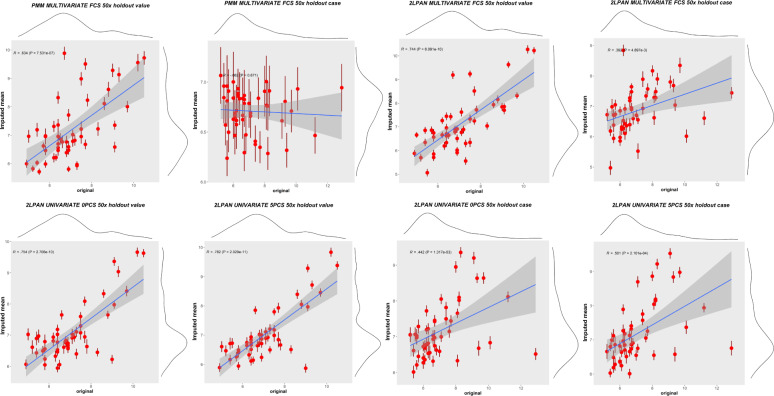
Over-imputation plot demonstrated the mean and standard error of imputed values for the observed 50 holdouts where imputed values would lie if they were missing in the GNSIS dataset using HbA1c as an example. The lm fit line with 95% CI was superimposed on the scatter plot. The Pearson’s correlation coefficient (*R*), as well as the significance of this correlation between 50 holdouts and imputed mean values were also present. This *R*-value represented the optimal correlation coefficient, which could be reached by each MI algorithm under multivariate or univariate setting. The kernel density plots at the margin of the scatter plot represented the corresponding distribution of observed and imputed 50 holdouts. Upper panels represent multivariate missing imputation using PMM or 2LPAN for HV or HC; Lower panels represent univariate missing imputation using 2LPAN with or without PCs derived from a comorbidity matrix as auxiliary variables.

The over-imputation plot (Fig. 7 for FCS and Supplementary Fig. 9 for Monotone) demonstrated the correlation between 50 holdouts and imputed mean values after 50 repeated MI. The *R*-value labeled in each panel represented the optimal correlation coefficient that could be reached by different imputation algorithms under different settings (multivariate or univariate missing).

Within the multivariate missing framework, our results showed that the 2l.pan outperformed PMM for this variable with a larger average Correlation Coefficient between imputed and holdout values under two simulated missingness patterns as shown in Table 2. The average *correlation coefficient (R)* was higher when using multivariate 2l.pan (e.g., *R* = 0.536 for 50 HVs using FCS) than multivariate PMM (e.g., *R* = 0.401 for 50 HVs using FCS), regardless of the imputation procedure (FCS or monotone) or simulation policy (HV or HC). Imputation performance slightly improved with increased average *R*, decreased variance (Standard Error) and coefficient of variance (CV) when using univariate 2l.pan including PCs that were derived from comorbidity information as latent variables (e.g., *R* = 0.473, SE = 0.012, CV = 0.179, compared to *R* = 0.462, SE = 0.014, CV = 0.214 for 50 HVs; *R* = 0.3, SE = 0.016, CV = 0.377, compared to *R* = 0.271, SE = 0.019, CV = 0.496, for 50 HCs). In all of our simulation experiments, HC consistently showed lower correlation (average *R*) and larger SE than HV, suggesting increased variance of imputation.

**Table 2:** Comparing the average correlation coefficient between imputed and holdout values of HbA1c from different imputation methods.

Imputation framework	Imputation method	Randomly holding-out 50 values					Randomly holding-out 50 complete cases				
		Correlation coefficient^a^	SE^b^	Coverage rate^c^	Average width^d^	Coefficient of variance^e^	Correlation coefficient	SE	Coverage rate	Average width	Coefficient of variance
Multivariate missing	PMM MONOTONE	0.390	0.013	0.88	0.027	0.236	0.039	0.021	0.84	0.043	3.807
	PMM FCS	0.401	0.014	0.86	0.029	0.247	−0.012	0.019	0.88	0.038	−11.196
	2LPAN MONOTONE	0.514	0.013	0.94	0.026	0.179	0.115	0.020	0.86	0.040	1.230
	2LPAN FCS	0.536	0.012	0.94	0.024	0.158	0.175	0.021	0.92	0.043	0.849
Univariate missing	2LPAN w/o auxiliary variables^f^	0.462	0.014	0.86	0.029	0.214	0.271	0.019	0.90	0.037	0.496
	2LPAN w auxiliary variables	0.473	0.012	0.84	0.025	0.179	0.300	0.016	0.84	0.033	0.377

*PMM* predictive mean matching, *FCS* fully conditional specification, *SE* standard error, *2LPAN* multi-level linear mixed model for imputation based on an assumption of homogenous within-group (i.e., patient ID) variances at level one data.

^a^Average of correlation coefficient (*R*) between imputed and holdout 50 values across the 50 complete sets.

^b^Standard error of correlation coefficient across the 50 complete sets.

^c^Coverage rate represents the proportion of confidence intervals (95%) that contains the value of correlation coefficient.

^d^Average width represents the average width of the confidence intervals (95%).

^e^Coefficient of variance for *R* is the ratio of standard deviation to the average *R*. This ratio represents the extend of variability (dispersion) relative to the average correlation coefficient.

^f^The auxiliary variables are the five main principal components derived from PCA of the comorbidity matrix.

## Discussion

The laboratory values in this study were collected from two different diseases cohorts, ischemic stroke, and heart failure, respectively, and data were acquired from the EHR from two large health care systems from different geographical areas with a distinct ethnic distribution. Using these datasets, our study (1) improved the understanding of missingness patterns in real-world EHRs, (2) assessed and compared the performance of commonly used imputation algorithms when applied to a broad range of laboratory variables, and (3) identified strategies for enhancing imputation performance by leveraging auxiliary information from patient’s comorbidity data.

Our analysis of quantitative laboratory variables from two datasets indicates an MNAR, which the margin plots were not able to show unless an in-depth knowledge of the cohort such as comorbidities was provided^10^. MNAR is a type of missing when the value of the variable that is missing is related to the reason it is missing, alternatively, the missingness is dependent on the missing values themselves given the observed data. MNAR was recognized in clinical trial data^16,27^ as well as EHR data from this study. Missingness in the repeated measurement in the clinical trial data is related to the patients’ responsiveness to the treatment, resulting in compliance and dropout issues. Similarly, the missingness in all common laboratory variables was related to individual disease burden in this study. This nonintentional missing was disguisable and other known (insurance, social-economic status, educational background) or unknown factors might also contribute to MNAR. Our analysis showed that the probabilities of missingness for all laboratory variables were related to disease burden. Patients with missing values are more likely to have a laboratory value within a normal range rather than within a range of observed data.

Our data also showed that when one test result was missing for a patient, other tests with a higher missingness rate for that patient were also likely to be missing, suggesting a “monotone” pattern of missingness. A “monotone” pattern may imply that missingness likely happens to a group of patients who do not seek health care regularly. Both datasets had a combination of monotone missingness and varying degrees of random missingness. The missing rates before and after the index date were similar in the case of HF; however, less missing after the index date compared to before the index date was observed in the GNSIS dataset (Fig. 2g, h). The difference between GNSIS and HF could be due, in part, to the higher mortality in HF, differences in social-economic status (e.g., insurance). Nonetheless, one should not assume that the rate of missingness will be always lower after a patient has an acute event.

Our simulation policy experiments (HC and HV) were designed to mimic different patterns of missingness in EHR. Using these simulations, we were able to identify experimental design strategies to improve the model performance and the stability of the nRMSE. To determine how many repeated imputations are necessary to reach an unbiased conclusion on the performance of commonly used MI algorithms, we evaluated nRMSE and compared the level and speed of the uncertainty propagated after 5, 10, 20, 30, 40, 50 repeated imputations. At the first 5 to 10 complete imputed sets, mean nRMSE from 2l.pan-FCS may not show a statistically significant difference from the mean from 2l.pan_MONOTONE; however, after 50 repeated imputations, the mean nRMSE reached a plateau for most of the laboratory variables in the HV design. However, in the HC design, the nRMSE error metric did not reach a plateau for some variables even after 50 repeats, irrespective of the imputation algorithms. The latter suggests that the uncertainty brought by MI was larger but propagated slower on the most informative cases when missingness was monotone. This observation corroborates that the monotone missingness in informative cases is the worst type of missingness, which translates to a lack of routine checkups or follow-up in at-risk patients.

Our simulation results indicate that the cross-sectional PMM may not be an optimal algorithm for a small dataset with a high proportion of missing values when compared to multi-level imputation (e.g., 2l.pan). The 2l.pan leverages both level 1 and 2 variables and allows switching regression imputation between level 1 and level 2 data^28^. In fact, in the HV simulation experiments, we observed that the 2l.pan showed better CR than PMM. The PMM algorithm was developed to provide a semi-parametric approach to imputation for settings where the normal distribution is not an appropriate assumption. Thus, PMM—as a mosaic form of donor-based and regression-based algorithms—was compared with the multi-level imputation. The uncertainty for missing values could be underestimated by PMM, resulting in poor coverage with increased variability of CR. This is because only a few similar observed cases were available for some variables with a high-level of missingness.

However, the advantage of multi-level over cross-sectional imputation was observed primarily in the GNSIS cohort. The lack of improvement in the HF cohort was likely because no substantial improvement in the percentage of subjects with at least one measure after the event was observed (see Fig. 2h). The multi-level imputation has limited ability to leverage post-event information to make a better prediction of pre-event missing values.

When comparing monotone to FCS imputation with the Monte Carlo iterative procedure, we always observed better performance with FCS. We also compared the cross-sectional imputation (PMM) to multi-level multivariate imputation such as 2l.pan (FCS-LMM) or 2 l.norm (FCS-LMM-het), which was based on an assumption of homogenous or heterogeneous within-group variances respectively^18,29^. Our analysis showed that when the imputed data was out of the normal range, higher variation may have increased the within- and between -imputation variance but did not improve the prediction accuracy. This leads to an important aspect in the utility of laboratory measurements; in most realistic clinical settings, a diagnosis is based on values that are outside of the normal range^23^.

Evaluating the performance gain when incorporating auxiliary information from patient’s comorbidity data was done by co-analyzing patient diagnosis patterns in conjunction with their laboratory measurements. We introduced PCs derived from PCA of comorbidity matrix to the multi-level univariate imputation algorithms such as 2l.pan. Using this design strategy we were able to add latent variables to the final prediction model^30^.

Including proper auxiliary variables mitigates the bias in maximum likelihood estimates caused by MAR or MNAR mechanism, particularly when an imputed variable and auxiliary variables are nonlinearly related^31^. Our result from univariate imputation by including PCs, as auxiliary (latent) variables, also reduced bias in the estimates. However, including auxiliary variables in the imputation may also increase the standard errors of the estimates substantially when the sample size is small, and the proportion of missing data is not trivial. Such an adverse effect may also occur when including some auxiliary variables to make the MAR assumption more plausible, especially when the auxiliary variables are not normally distributed^31^. Finally, when the outcome variable is the outcome of interest in the analytic model, this variable is highly recommended to be included in the imputation model to improve the performance in the analytic model^32^.

The strengths of this study lie in the followings: (1) Description of the missing pattern and exploration of the mechanism of missing in the laboratory variables of EHR database; (2) Simulation of two missingness patterns recognized in this study—monotone and arbitrary missingness; (3) Comparative assessment of well-established commonly used cross-sectional and multi-level imputation methods, integrated with two imputation procedures (monotone or FCS); (4) Use of latent information extracted from comorbidity matrix as auxiliary variables in the imputation model; and (5) Evaluation of the generalizability of the findings by analyzing two datasets with distinct disease cohorts from two different healthcare systems. Furthermore, the phenotype definition for each of the conditions was carefully assessed and validated in the previous publications^33–36^.

Since simulating the missingness pattern of laboratory variables in EHR is challenging, the pattern of missingness and imputation strategy used in this study may not apply directly to other diseases or datasets. Furthermore, imputation of missingness was based on using quantitative variables and may not translate to categorical variables or derived/modified variables (ratio, converted values). Given that our understanding of realistic missing patterns is still limited, in this study only two simulated missingness scenarios were evaluated and these patterns may not occur or fully represent the pattern of missingness in realistic settings. Finally, multiple MI methods including FCS, joint model (JM), EM-based algorithms, and their extended forms, were also applied to longitudinal and clustered data^29,32^. As our goal was to align ourselves with current standard practices in EHR-mining, our study did not include all regression-based algorithms, JM, or EM-based algorithms.

As future directions, we are exploring how the inclusion of the auxiliary variables affects the bias and precision of the imputation models. In this analysis, we are assessing the various parameters such as the cohort sample size, number of imputations, missing rate, number of iterations, as well as the correlation between variables. The EHR dataset could also be nested hierarchically by the healthcare center. Having a healthcare center as an additional level of data clustering will be considered in the multi-level imputation model, especially when data from different centers are pooled together for analysis. Finally, our study is part of a larger effort to improve risk stratification for heart failure and ischemic stroke, using machine-learning applied to data from EHRs.

In conclusion, the pattern of missingness in EHR laboratory variables was *not random* and was highly associated with patients’ comorbidity data. Multi-level imputation (2l.pan) showed smaller nRMSE for most variables compared to cross-sectional methods. MI with Markov Chain iterations such as FCS performed better than the monotone procedure. In the case study of HbA1c, univariate imputation using a multi-level model with FCS, which leveraged comorbidity as latent variables in the imputation, had superior performance compared to the same method without these auxiliary variables.

Finally, the missing pattern and mechanism for a given dataset should first be recognized. Whether the competition is favoring a certain method or procedure has to be determined in the “real-world” data with “real-world” missingness by considering recognized and unrecognized missing pattern/mechanism, as well as the plausible distribution of missing data. Our study provides benchmarking and practice recommendations based on common algorithms for imputing laboratory variables if these variables follow similar missingness patterns.

## Methods

This study was approved by both Geisinger and Sutter Health Institutional Review Board and a waiver of consent was granted because of using de-identified EHR data. Ordered and resulted laboratory tests completed within the index date ± 2 years for Sutter Health Heart Failure (HF) or index date ± 3 years for “Geisinger NeuroScience Ischemic Stroke (GNSIS)” were used for imputation, where the index date was defined as the first time the disease of interest (i.e., ischemic stroke or heart failure) meet the diagnosis criteria^33–36^. Only quantitative laboratory values were considered for imputation. Similar to a moving time window and stepwise regression procedure^37,38^, the last valid observation before and the first observation after the index date were extracted from the corresponding time blocks. Imputation of missing values in each laboratory variable was based on the information of observed values from this and other laboratory variables. We first assessed the missing pattern between variables, time blocks, or cohorts. We studied two missing patterns by randomly holding-out 50 laboratory values (HV) and 50 complete patient records (HC). To mimic Missing-completely-at-random (MCAR) or Missing-at-random (MAR) we used the HV and to mimic monotone missing we used the HC simulation. We selected commonly used error metrics, to assess the performance of the algorithms. In a case study, we imputed hemoglobin A1c with and without comorbidity-derived latent information to evaluate the utility of auxiliary variables in a univariate imputation framework.

### Data sources

Two distinct datasets were used: the GNSIS cohort^33–35^ and the Sutter Health heart failure cohort (HF)^36^. All investigators in this study had no control of missingness in EHR data collection.

The GNSIS database is composed of EHR data for patients with well-defined ischemic stroke from September 2003 to May 2019^33,34^, The ICD-9-CM/ICD-10-CM diagnostic criteria for phenotypes were previously published^34,35^. The comorbidity information based on ICD-9-CM or ICD-10-CM diagnosis was extracted within index data ±3 years. Comorbidity was defined as a qualified diagnosis associated with either two outpatient visits or one inpatient visit. The entire laboratory data, based on Logical Observation Identifiers Names and Codes (LOINC), for the cohort, were extracted and included in this study.

The Sutter Health HF database includes incidence heart failure cases identified from Sutter Health primary care population^36^. Longitudinal EHR data were extracted on incidence cases diagnosed between January 1, 2010, to December 31, 2017. Encounter-based laboratory results with the corresponding LOINC identifiers within a 2-year window before or after the index date were extracted. For the diagnosis domain, all ICD10 codes had been converted to ICD9 codes first. ICD-9 codes from outpatient office visits or phone visits were grouped using Clinical Classifications Software (CCS) [https://www.hcup-us.ahrq.gov/toolssoftware/ccs/ccs.jsp]. The CCS level 3 was adopted to group 5379 ICD-9 codes into 363 unique CCS groups.

To minimize data sparsity, ICD and CCS codes were only used if they were observed in at least 20% of the patients.

### Recognition of missing pattern and mechanism

Missing values were defined as either not tested or tested but with values outside of the three interquartile range (IQR). Analysis of missingness was limited to laboratory variables (see Supplementary Table 1) where the proportion of missingness was <75%^39^.

We created a fluxplot^30^ to capture the relationship between variables. In particular, the fluxplot can facilitate the identification of the relationship of missing and observed data between variables using influx and outflux coefficients and the tradeoff between them. The influx coefficient (*I*_*j*_) of a variable quantifies how well the missing data is connected to the observed data on other variables (see Eq. (1)); the outflux coefficient (*O*_*j*_*)* of a variable quantifies how well the observed data is connected to the missing data on other variables^17^ (see Eq. (2)). In general, variables that are located closer to the sub-diagonal tend to be better connected than those farther away.

The *influx coefficient I*_*j*_ is defined as^30^

1 $$I_j = \frac{{\mathop {\sum }\nolimits_j^p \mathop {\sum }\nolimits_k^p \mathop {\sum }\nolimits_i^n r_{ij}\left( {1 - r_{ij}} \right)r_{ik}}}{{\mathop {\sum }\nolimits_k^p \mathop {\sum }\nolimits_i^n r_{ik}}}$$

The coefficient is equal to the number of variable pairs (*Y*_*j*_,*Y*_*k*_) with *Y*_*j*_ missing and *Y*_*k*_ observed, divided by the total number of observed data cells. *R* is an *n* by *p* matrix filled with 0 or 1 as *a response indicator*. *Y* and *R* are denoted by *y*_*ij*_ and *r*_*ij*_, respectively, where subject index *i* = 1, 2, …, *n* and variable index *j* = 1, 2, …, *p*. If *y*_*ij*_ is observed, then *r*_*i* *j*_ = 1, and if *y*_*ij*_ is missing, then *r*_*ij*_ = 0. So did *r*_*ik*_.

The *outflux coefficient O*_*j*_ is defined in an analogous way as^30^

2 $$O_j = \frac{{\mathop {\sum }\nolimits_j^p \mathop {\sum }\nolimits_k^p \mathop {\sum }\nolimits_i^n r_{ij}(1 - r_{ik})}}{{\mathop {\sum }\nolimits_k^p \mathop {\sum }\nolimits_i^n 1 - r_{ij}}}$$

The quantity *O*_*j*_ is the number of variable pairs with *Y*_*j*_ observed and *Y*_*k*_ missing, divided by the total number of incomplete data cells.

We explored the pattern of missingness by the Rubin^40^ classification—Missing-completely-at-random(MCAR), Missing-at-random(MAR), and Missing-not-at-random (MNAR). We used the margin plot (Supplementary Fig. 2) to capture the missingness pattern between “before the index date” and “after the index date” or between two laboratory variables.

### Simulation of missingness

For holdout values (HV), the randomly selected 50 holdout values per variable came solely from observed data, thus the data were MAR. The probability of being missing was the same for all cases when the selection of 50 holdouts was made by a random pick from a variable without missing value. This variable was said to be MCAR. Thus, HV represented MAR or MCAR, defined by Rubin^40^ and others^30^.

For holdout cases (HC), we held out entire laboratory values for 50 cases, which were randomly selected from all complete cases. Under HC, we maintained the sequence of the missing level across all variables and kept the original connection between missingness in one variable and observation in the other variable throughout the dataset except for holdout cases. The simulation of missingness created using this procedure reflected the theory of monotone missingness^41^, namely ordering one laboratory test was dependent upon other tests, or missing in other variables resulted in missing in one variable.

### Imputation strategy

*Monotone Multiple Imputation:* Multiple imputation (MI) is featured by a missing measure to be imputed multiple times. We utilized the latest implementation of the monotone MI algorithm in MICE^30,41^ to impute each missing value, where a missing pattern is said to be “monotone” if the variables *Y*_*j*_ (*j* = 1, 2, …, *k*) can be ordered such that if *Y*_*j*_ is missing then all variables *Y*_*−j*_ with *k* > *j* are also missing.

*The procedure for multivariate monotone imputation* ^30^

1. Create a short format of GNSIS or HF dataset and choose a single level (PMM) or multilevel (2L.PAN) imputation models;
2. Sort from low to high for *p* incomplete variables (*j* = 1, 2, …, *p*) according to the frequency of missingness, *Y* denotes the *n* by *p* matrix containing the data values on *p* variables for all *n* units in the sample; $Y_j^{{\mathrm{obs}}}$ represents a vector of observed value for the *j* variable;
3. Draw temporary parameter *ϕ*_1_ ($\dot \phi _1$) from a univariate conditional density function, *P*($Y_1^{{\mathrm{obs}}}$| *X*), where *X* represents the completely observed covariates such as *TIME, SEX*;
4. Impute temporary *Y*_1_ ($\dot Y_1$) based on *P*($Y_1^{{\mathrm{mis}}}$|*X*,$\dot \phi _1$);
5. Draw $$\dot \phi _2$$∼*P*($$Y_2^{{\mathrm{obs}}}$$| *X*,$$\dot Y_1$$);
6. Impute $$\dot Y_2\sim P$$($$Y_1^{{\mathrm{mis}}}$$|*X*,$$\dot Y_1$$,$$\dot \phi _2$$);
7. ⋮⋮;
8. Draw $$\dot \phi _p$$∼*P*($$Y_p^{{\mathrm{obs}}}$$|*X*,$$\dot Y_1$$ *, …*, $$\dot Y_{p - 1}$$);
9. Impute $$\dot Y_p\sim P$$($$Y_p^{{\mathrm{mis}}}$$|*X*,$$\dot Y_1$$*, …*,$$\dot Y_{p - 1}$$,$$\dot \phi _p$$);
10. Repeat steps 3–9 for *m* – 1 times to obtain *m* complete sets.
11. (optional) apply to the analysis model (LMM) and calculate estimates (exponentiate) and variance.
12. (optional) Combine the results by Rubin’s rule to obtain mean estimates (exponentiate), variance (including within-imputation variance and between-imputation variance)

Note: this algorithm not only incorporates the uncertainty due to deviations around the regression line (step 3) but also reflects the variation of the regression line itself due to finite sampling (step 8).

#### Fully conditional specification

Fully conditional specification (FCS), also known as chained equations and sequential regressions, is an iterative Markov Chain method that can be used when the pattern of missing data is arbitrary or a mixture of monotone and arbitrary. FCS draws missing values iteratively from a specified set of conditional probabilistic distributions, $P(Y_j|X,Y_{ - j},R,\phi _j)$^30^, compared to monotone imputation with a fixed sequence of MI. When applying this iterative procedure to update the parameters (intercept, slope, and error) for a given number of iterations (for instance, *n* = 500), one imputed complete set is generated. When the entire process of imputation has been repeated *m* *–* *1* times, *m* imputed complete sets are reached. Therefore, MI can help “fill in” the missing data with plausible values by adding variability to the analyses— facilitating parameter estimation for each incomplete variable.

*The procedure for multivariate FCS imputation* ^30^

1. Create a short format of GNSIS or HF dataset and choose a single level (PMM) or multilevel (2L.PAN) imputation model;
2. Specify an imputation model *P*($Y_j^{{\mathrm{mis}}}$| $Y_j^{{\mathrm{obs}}},Y_{ - j}$*, R*, *ϕ*_*j*_) for *Y*_*j*_ with variable index *j* = 1, 2, …, p without sorting the sequence of variables by frequency of missingness, *Y*_−*j*_ represents other variables but not *j* variable; *R* is a n by p matrix filled with 0 or 1 as *response indicator*. The elements of *Y* and *R* are denoted by *y*_*ij*_ and *r*_*ij*_, respectively, where subject index *i* = 1, 2, …, *n* and variable index *j* = 1, 2, …, *p*. If *y*_ij_ is observed, then *r*_*ij*_ = 1, and if *y*_*ij*_ is missing, then *r*_*ij*_ = 0. *ϕ*_*j*_ is unknown regression model parameters for j variable (see Schafer et al. for PAN model parameter)^42^; *t* represents *t* number of MCMC iterations.
3. For each *j*, fill in starting imputations $Y_j^{ \cdot 0}$ by random draws from $Y_j^{{\mathrm{obs}}}$ and fill in starting value for *ϕ*^0^ by Gibbs sampler in MCMC procedure.
4. For *t* ← 1 to *N*. *N* is 500 burn-in iterations
5. Repeat
6. For *j* ← 1 to *p*. *p* is 45 or 38 for GNSIS or HF respectively.
7. Define $\dot Y_{ - j}^t$ = ($\dot Y_1^t$, …, $\dot Y_{j - 1}^t$, $\dot Y_{j + 1}^{t - 1}$, …, $\dot Y_p^{t - 1}$) as the currently complete data expect Y_*j*_;
8. Draw $\dot \phi _j^t$ ~ *P*($\phi _j^t$|$Y_j^{{\mathrm{obs}}}$, $\dot Y_{ - j}^t$, *R*); This is a step to get a new regression model parameter.
9. Draw imputations $\dot Y_j^t$ ~ *P*($Y_j^{{\mathrm{miss}}}$|$Y_j^{{\mathrm{obs}}}$, $\dot Y_{ - j}^t$, *R*,$\dot \phi _j^t$). This is imputation step
10. End repeat *j*
11. End repeat *t*
12. Repeat steps 3–9 for *m* – 1 times to obtain *m* complete sets.
13. (optional) apply to analysis model (linear mixed-effects regression model) and calculate estimates (exponentiate) and variance.
14. (optional) Combine the results by Rubin’s rule to obtain mean estimates (exponentiate), variance (including within-imputation variance and between-imputation variance)

We chose predictive mean matching (PMM) as the benchmark method for the cross-sectional imputation of continuous variables because it is a hot-deck method, where values are imputed using existing values from the complete cases matched with respect to some metric^15^. In this study, we used Type 1 matching with a Bayesian β and a stochastic matching distance^30^. For each missing value, PMM finds a set of observed values (e.g., five donors) from all complete cases that have predicted values closest to the predicted value for the missing entry and considers the donor with the closest predicted mean as the imputed value for that missing entry. Therefore, imputed values from PMM are restricted to the observed values. For this PMM-FCS approach, we also evaluated the mean and standard deviation for each laboratory value after each round of iteration (*n* = 10 for GNSIS; *n* = 15 for HF due to a higher level of missingness) to ensure statistical convergence. However, the Monte Carlo iterative procedure does not apply to monotone imputation. For FCS, the default iteration of 500 was selected. We utilized the latest implementation of the PMM-FCS and PMM-MONOTONE algorithms in MICE^30,41^.

#### Multi-level multivariate missing imputation

EHR data can be regarded as multi-level time-series data. We considered the repeated measure at the individual level as level one data (see below “Level one model” in Eq. (3)). The covariates such as *TIME* (i.e., before or after the index date, which was dummy coded) can be treated either as level one (Level one model) or both level one and level two (i.e., a random intercept in Eq. (4) and a random slope in Eq. (5)).

We used the MICE 2l.pan^42^ or 2l.norm^43^ for the imputation based on an assumption of homogenous or heterogeneous within-group (i.e., patient ID) variances in level one data, respectively^43^. We defined the cluster variable (*C*) as “ID”. We compared the two-level model to the cross-sectional PMM model to determine if there is any significant improvement in the prediction of missingness with this mixed model.

*Level one model:*

3 $$\begin{array}{l}LAB_{jc}^{{\mathrm{miss}}} = \beta _{0c} + \beta _{1c}TIME + \beta _{jc}LAB_{jc}^{{\mathrm{obs}}} + \beta _{ - jc}LAB_{ - jc} \ldots + \varepsilon _{ic};\\ \varepsilon _{ic}\sim N\left( {0,\delta _\varepsilon ^2} \right),LAB \in \left( {LAB_j,j = 1,2, \ldots ,p} \right)\end{array}$$

*Level two model with a random intercept:*

4 $$\begin{array}{l}\beta _{0c} = \alpha _{00} + u_{0c};\\ u_{0c}\sim N\left( {0,\delta _{u_0}^2} \right)\end{array}$$

*Level two model with a random intercept and a random slope for TIME (optional):*

5 $$\begin{array}{l}\beta _{0c} = \alpha _{00} + \alpha _{01}TIME + u_{0c};\\ u_{0c}\sim N\left( {0,\delta _{u_0}^2} \right)\end{array}$$

where, *ε*_*ic*_ is a value drawn from a Normal random vector with mean = 0, variance = $\delta _\varepsilon ^2$ for the imputed variable *j* in each cluster (*C*); *β*_*0c*_ represents the constant value of intercept with additional random error for a random intercept model. *β*_*0c*_ *=* *α*_*00*_ *+* *u*_*0c*_ represents a constant intercept modified by a random error, *u*_*0c*_, which is a value drawn from a Normal random vector with mean = 0, variance = $\delta _{u_0}^2$ for a random intercept in each cluster (*C*); and *β*_*-jc*_*LAB*_*-jc*_ represents a group of additive terms derived from variables but not the *j* variable in each cluster (*C*) in a stochastic linear regression model. Some variables, e.g., *TIME*, can have both fixed (*β*_*1c*_) as well as a random effect (*α*_*01*_*)* in the multi-level imputation model. Thus, *β*_*0c*_ represents a fixed intercept with random error plus a random slope for *TIME*.

#### Multi-level univariate missing imputation

The multi-level univariate imputation was considered as an alternative approach only when one continuous variable was assumed to have missing values (univariate missing data). The comorbidity information (in the form of CCS for HF cohort or ICD for the GNSIS cohort) was used in the principal component analysis (PCA). Based on the scree plot, the major five PCs, which explained more than 60% of the variance, were selected as auxiliary variables for the univariate imputation.

We applied multi-level univariate imputation to each missing lab value at a time, along with the PCs extracted from the comorbidity matrix. Similar to the above multivariate imputation, this method can have a level one model (Eq. (6)) and level two model (a random intercept in Eq. (7) and a random slope in Eq. (8)).

Level one model for incomplete quantitative variables:

6 $$\begin{array}{l}LAB_{jc}^{{\mathrm{miss}}} = \beta _{0c} + \beta _{jc}LAB_{jc}^{{\mathrm{obs}}} + \beta _{1c}TIME + \beta _{2c}PC1\\ + \beta _{3c}PC2 + \beta _{4c}PC3 + \beta _{5c}PC4 + \beta _{6c}PC5 + \varepsilon _{ic};\\ \varepsilon _{ic}\sim N\left( {0,\delta _\varepsilon ^2} \right),LAB \in \left( {LAB_j,j = 1,2, \ldots ,p} \right)\end{array}$$

*Level two model with a random intercept:*

7 $$\begin{array}{l}\beta _{0c} = \alpha _{00} + u_{0c};\\ u_{0c}\sim N\left( {0,\delta _{u_0}^2} \right)\end{array}$$

*Level two model with a random intercept and a random slope for TIME (optional):*

8 $$\begin{array}{l}B_{0c} = \alpha _{00} + \alpha _{01}TIME + u_{0c};\\ u_{0c}\sim N\left( {0,\delta _{u_0}^2} \right)\end{array}$$

Where, all *βs* are estimates based on complete cases; *ε*_*jc*_ is determined by the variance of the residual *ε*, which can be a random draw from the set of sample residuals for the complete cases with mean = 0, variance = $\delta _\varepsilon ^2$ for the imputed variable *j* in each cluster (*C*); *β*_*0c*_ represents the constant value of intercept with additional random error for a random intercept model. *α*_*00*_ *+* *u*_*0c*_ represents a constant intercept modified by a random error, *u*_*0c*_, which is a value drawn from a Normal random vector with mean = 0, variance = $\delta _{u_0}^2$ for a random intercept in each cluster (*C*); Some variables, e.g., *TIME* can have both fixed (*β*_*1c*_) as well as random effect (*α*_*01*_*)* in the multi-level imputation model. Thus, *β*_*0c*_ represents fixed intercept with random error plus *TIME* with a random slope.

### A case of hemoglobin A1c

HbA1C has been included as one of the major predictive variables in many diagnostic and prognostic models for cardiometabolic diseases and related complications. High-level of missingness in HbA1C in EHR limits its application in the prediction model due to the sample size. The inclusion of imputed HbA1C in the prediction model for the post-ischemic stroke mortality has shown to be important in our previous study using the GNSIS dataset^44^. Missing hemoglobin A1c (HbA1c) was imputed by the Multi-level multivariate imputation approach as well as the multi-level univariate imputation approach where the comorbidities were taken as latent variables. HbA1c has been connected to other metabolic diseases (comorbidities)^45^ and could be an ideal laboratory variable for univariate imputation using PCs from the comorbidity matrix as latent variables.

### Model evaluation

In both HV and HC experiments, we heldout 50 observed values for each laboratory variable before the index date and calculate the errors between observed and predicted values. We repeated each process up to 50 times and calculated the mean, standard error (SE), and 95% confidence interval of predicted values for each holdout and calculated the coverage rate (CR) and average width (AW). We used normalized RMSE (nRMSE) to ensure this error metric was on the same scale across different laboratory variables. The stability of the mean and SE of nRMSE, which reflected the propagation of uncertainty in those imputed holdouts after a sequential number of MI, were also assessed. Levene’s test was utilized to determine an equal variance of the nRMSE from two compared imputation algorithms, e.g., 2l.pan-FCS and PMM-FCS. Shapiro–Wilk test was applied for the normality test of the difference of nRMSE for each comparison. The nRMSEs derived from the different algorithms were compared using an unpaired *t*-test with Bonferroni correction for multiple tests. The algorithm that resulted in the smallest RMSE was the optimal approach for that laboratory variable.

The evaluation metrics include the following measures:

1. Root mean square error (RMSE)—RMSE penalizes the larger errors and is sensitive to extreme values. We normalized RMSE by standard deviation, *δ* (See Eq. (9)).
  9 $$n{\mathrm{RMSE}} = \sqrt {\frac{{\mathop {\sum }\nolimits_{i = 1}^n \left( {\hat Y_i - Y_i} \right)^2}}{n}} \delta ^{ - 1}$$
  Note: *Y*_*i*_ represents holdout values and $\hat Y_i$ is the corresponding imputed value
2. Coverage rate (CR)—CR represents the proportion of confidence intervals that contain the imputed value. We calculated the mean of CR for each subject after 50 repeated imputations.
3. Average width (AW)—AW represents the average width of the confidence intervals and is an indicator of statistical efficiency. We calculated the mean AW for each subject after 50 repeated imputations.

Over-imputation scatter plots for each laboratory variable are generated as graphical diagnostic tools^46^ to assess the suitability of different imputation algorithms.

### Reporting summary

Further information on research design is available in the Nature Research Reporting Summary linked to this article.

## Supplementary information

Supplementary Information

Reporting Summary

## Data Availability

The data analyzed in this study is not publicly available due to privacy and security concerns. The data may be shared with a third party upon execution of data sharing agreement for reasonable requests, such requests should be addressed to V. Abedi or S.Y.

## Code availability

Codes and additional meta-data, summary plots, and information can be found at

https://github.com/TheDecodeLab/Imputation-LaboratoryValues-EHR_v2.0.
