# Supplementary material for: Imputation of missing values for electronic health record laboratory data
Source: NPJ Digit Med. 2021 Oct 11;4:147. doi: 10.1038/s41746-021-00518-0 (PMC8505441; doi:10.1038/s41746-021-00518-0)
Supplement: Supplementary file 1 — Supplementary Information [file 41746_2021_518_MOESM1_ESM.pdf]

Supplementary Table 1. Description of common laboratory variables in Geisinger as well as Sutter Health EHR for patients with ischemic stroke (1A) in GNSIS dataset or heart failure (1B) in HF dataset respectively.

1A

GNSIS(Geisinger)										
Miss% before	Miss% after	LAB_INDEX	LONIC_ID	COMPONENT	REFERENCE LOW	REFERENCE HIGH	UNIT	Mean(SD) before	Mean(SD) after	Corr Coefficient (before:after)
35.89	2.4	X2823	2823-3	Potassium	3.5	5.1	MMOL/L	4.256(0.481)	4.008(0.447)	0.328
36.08	2.26	X17861	17861-6	Calcium	8.4	10.2	MG/DL	9.272(0.55)	8.924(0.522)	0.416
36.1	2.14	X2028	2028-9	Carbon dioxide	22	32	MMOL/L	26.834(3.035)	25.446(3.121)	0.467
36.18	2.25	X2075	2075-0	Chloride	98	107	MMOL/L	101.975(3.925)	103.781(4.128)	0.507
36.51	2.37	X2951	2951-2	Sodium	135	146	MMOL/L	139.234(3.318)	139.361(3.181)	0.461
37.16	4.02	X3094	3094-0	Urea nitrogen	6	20	MG/DL	19.239(8.073)	17.617(7.76)	0.622
38.41	6.16	X2160	2160-0	Creatinine	0.6 (0.5)	1.2 (1.0)	MG/DL	1.009(0.325)	0.954(0.321)	0.804
38.77	4.32	X2345	2345-7	Glucose	70	120	MG/DL	119.727(38.239)	120.475(36.528)	0.452
39.26	2.61	X718	718-7	Hemoglobin	12	15.3	G/DL	13.081(1.943)	12.965(1.891)	0.728
39.75	2.68	X4544	4544-3	Hematocrit	36.0 (40.0)	45.2 (48.4)	%	39.048(5.377)	38.556(5.178)	0.698
39.89	2.79	X789	789-8	Erythrocytes	3.85 (4.50)	5.15 (5.25)	M/UL	4.329(0.637)	4.289(0.61)	0.731
39.95	2.8	X786	786-4	Erythrocyte mean corpuscular hemoglobin concentration	32	36	G/DL	33.425(1.239)	33.597(1.19)	0.699
40.1	2.85	X32623	32623-1	Platelet mean volume	6.6	11.1	FL	9.854(1.397)	9.746(1.328)	0.752
40.12	3.07	X777	777-3	Platelets	140	400	K/UL	232.984(75.418)	216.817(68.57)	0.723
40.12	3.07	X787	787-2	Erythrocyte mean corpuscular volume	81.5	97.5	FL	90.546(5.439)	90.162(5.324)	0.861
40.17	3.18	X785	785-6	Erythrocyte mean corpuscular hemoglobin	27	34	PG	30.294(2.113)	30.323(2.084)	0.876
40.28	4.11	X6690	6690-2	Leukocytes	4	10.8	K/UL	8.079(2.665)	8.559(2.939)	0.46
40.66	4.26	X10466	10466-1	Anion gap 3	7	15	MMOL/L	10.515(3.218)	10.17(3.17)	0.443
41.36	4.43	X788	788-0	Erythrocyte distribution width	11.5	15.5	%	13.944(1.307)	13.837(1.246)	0.77
46.52	25.1	X1743	1743-4	Alanine aminotransferase	10	35 (50)	U/L	21.683(10.84)	22.661(12.121)	0.453
46.8	20.49	X61151	61151-7	Albumin	3.8	5	G/DL	3.941(0.501)	3.75(0.517)	0.535
47.53	8.75	X736	736-9	Lymphocytes/100 leukocytes	18	42	%	22.22(9.565)	21.644(10.113)	0.503
47.63	22.66	X2885	2885-2	Protein	6	8.3	G/DL	6.874(0.647)	6.659(0.673)	0.448
47.67	9.11	X5905	5905-5	Monocytes/100 leukocytes	1	11	%	8.486(2.844)	8.43(2.756)	0.413
48	9.45	X742	742-7	Monocytes	0	1.1	K/UL	0.681(0.276)	0.71(0.289)	0.451
48.11	9.25	X731	731-0	Lymphocytes	1	4.8	K/UL	1.726(0.777)	1.732(0.778)	0.628
48.28	26.2	X30239	30239-8	Aspartate aminotransferase	10	35	U/L	24.519(8.921)	25.53(9.762)	0.385
48.48	9.9	X706	706-2	Basophils/100 leukocytes	0	2	%	-7.679(10.008)	-8.652(10.205)	0.402
48.71	24.42	X6768	6768-6	Alkaline phosphatase	0	153	U/L	81.756(27.609)	78.844(27.251)	0.672
48.76	10.05	X770	770-8	Neutrophils/100 leukocytes	40	75	%	65.687(11.447)	66.926(12.089)	0.412
48.94	25.06	X1975	1975-2	Bilirubin	0	1.2	MG/DL	0.531(0.291)	0.577(0.314)	0.55
51.3	15.77	X711	711-2	Eosinophils	0	0.7	K/UL	-0.828(0.388)	-0.918(0.426)	0.381
51.34	20.02	X713	713-8	Eosinophils/100 leukocytes	0	6	%	0.325(0.347)	0.254(0.385)	0.353
53.09	16.73	X704	704-7	Basophils	0	0.2	K/UL	-1.452(0.296)	-1.497(0.292)	0.517
53.62	16.6	X2093	2093-3	Cholesterol	NA	NA	MG/DL	178.579(46.356)	166.091(45.681)	0.597
54	16.76	X2085	2085-9	Cholesterol.in HDL	NA	NA	MG/DL	48.05(14.786)	44.925(14.348)	0.72
55.14	18.46	X13457	13457-7	Cholesterol.in LDL	0	129	MG/DL	99.853(38.611)	92.945(38.177)	0.586
55.28	17.9	X2571	2571-8	Triglyceride	0	150	MG/DL	142.896(70.842)	131.657(67.64)	0.603
55.47	19.43	X9830	9830-1	Cholesterol.total/Cholesterol.in HDL	NA	NA	RATIO	3.958(1.393)	3.948(1.433)	0.683
56.27	38.43	X751	751-8	Neutrophils	*NA* (1500)	*NA* (7800)	X 10^3 (cells/UL)	5.451(2.424)	5.866(2.736)	0.372
61.62	36.73	X50560	50560-2	Urine pH	5	7.5	UNITS	6.009(0.79)	6.034(0.773)	0.189
62.75	44.23	X3016	3016-3	Thyrotropin	0.27	4.2	MIU/L	2.249(1.485)	2.311(1.6)	0.478
67.83	29.91	X5902	5902-2	Coagulation tissue factor induced	11.5	14.6	SECONDS	14.233(1.839)	14.379(1.642)	0.456
68.32	30.59	X6301	6301-6	Coagulation tissue factor induced.INR	0.84	1.14	*NA*	1.108(0.173)	1.124(0.161)	0.45
71.09	40.69	X17856	17856-6	Hemoglobin A1c/Hemoglobin.total	4	5.6	%	7.025(1.633)	6.489(1.502)	0.743

Supplementary Table 1. Description of common laboratory variables in Geisinger as well as Sutter Health EHR for patients with ischemic stroke (1A) in GNSIS dataset or heart failure (1B) in HF dataset respectively.

1B

HF(Sutter Health)										
Miss% before	Miss% after	LAB_INDEX	LONIC_ID	COMPONENT_NAME	REFERENCE LOW	REFERENCE HIGH	UNIT	Mean(SD) before	Mean(SD) after	Corr Coefficient (before:after)
58.09	60.39	X547	2823-3	K+	3.5	5.1	MMOL/L	4.262(0.463)	4.281(0.484)	0.391
58.44	60.42	X546	2951-2	NA+	135	145	MMOL/L	139.45(3.184)	139.271(3.384)	0.473
58.46	60.52	X552	17861-6	CA	8.2	10.2	MG/DL	9.004(0.481)	9.031(0.466)	0.448
58.49	60.49	X548	2075-0	CL-	98	107	MMOL/L	103.437(3.727)	103.003(3.982)	0.514
58.49	61.06	X15715	2160-0	CREATININE	0.6	1.3	MG/DL	1.06(0.369)	1.147(0.434)	0.723
58.51	60.49	X541	2028-9	TCO2	21	32	MMOL/L	27.806(3.038)	28.081(3.177)	0.494
59.02	61.13	X550	3094-0	BUN	6	25	MG/DL	21.187(9.096)	23.187(10.671)	0.599
59.59	61.89	X549	2345-7	GLU	70	100	MG/DL	113.58(33.589)	113.365(32.696)	0.413
62.97	65.63	X513	718-7	HEMOGLOBIN	13.5 (11.7, 12.0)	18.0 (15.5, 15.5)	G/DL	12.836(1.821)	12.614(1.804)	0.637
63.04	65.66	X514	4544-3	HEMATOCRIT	40.0 (35.0)	52.0 (47.0)	%	39.292(5.078)	38.786(4.992)	0.617
63.23	65.78	X517	786-4	MCHC	31	36	G/DL	32.648(1.233)	32.468(1.258)	0.61
63.24	65.8	X512	789-8	RBC	4.40 (3.9)	6.00 (5.4)	M/UL	4.304(0.587)	4.24(0.575)	0.664
63.4	65.85	X515	787-2	MCV	80	100	FL	91.673(5.859)	91.888(6.06)	0.78
63.46	66.76	X554	1751-7	ALB	3.2	4.7	G/DL	3.603(0.435)	3.611(0.387)	0.495
63.46	66.09	X519	777-3	PLATELET COUNT	150	400	K/UL	228.131(70.929)	226.625(74.628)	0.66
63.57	66.1	X511	6690-2	WBC	4	11	K/UL	7.475(2.424)	7.341(2.306)	0.518
63.57	65.95	X516	785-6	MCH	27	33	PG	29.946(2.129)	29.856(2.323)	0.778
63.77	67.69	X564	1742-6	ALT	15	65	U/L	32.903(13.526)	30.757(13.142)	0.412
63.84	66.19	X518	788-0	RDW	<16.4		%	14.292(1.332)	14.526(1.486)	0.571
64.7	68.15	X553	2885-2	TP	6.4	8.2	G/DL	7.153(0.585)	7.173(0.584)	0.525
64.87	68.22	X557	1920-8	AST	0	37	U/L	23.651(9.351)	23.399(8.971)	0.43
65.21	68.47	X556	6768-6	AP	26	137	U/L	90.617(29.471)	91.361(32.13)	0.631
65.22	68.89	X555	1975-2	TBIL	<1.1		MG/DL	0.554(0.261)	0.571(0.259)	0.566
65.71	68.71	X754	706-2	BASEPHIL%	0	2	%	0.486(0.553)	0.492(0.556)	0.398
65.73	68.69	X624	770-8	NEUTROPHIL%	49	74	%	63.234(10.551)	63.738(10.894)	0.436
65.75	68.71	X522	736-9	LYMPHOCYTE%	26	46	%	23.995(9.045)	23.283(9.288)	0.518
65.78	68.76	X625	5905-5	MONOCYTE%	2	12	%	9.19(2.693)	9.249(2.84)	0.487
66.02	69.11	X753	713-8	EOSINOPHILS%	0	5	%	2.886(1.927)	2.947(2.026)	0.454
66.71	68.88	X813	742-7	MONOTYPE ABS	0	0.8	K/UL	0.672(0.253)	0.662(0.245)	0.513
66.76	68.98	X814	711-2	EOSINOPHILS ABS	0	0.5	K/UL	0.209(0.145)	0.21(0.149)	0.506
66.76	68.88	X812	731-0	LYMPHOCYTE ABS	1	5.1	K/UL	1.698(0.666)	1.624(0.67)	0.633
66.88	72.73	X598	2093-3	CHOLESTEROL	<200		MG/DL	164.172(42.202)	159.966(42.207)	0.59
66.9	69.08	X811	751-8	NEUTROPHIL ABS	2	8	X 10^3	4.783(1.992)	4.725(1.897)	0.401
67	72.72	X600	2085-9	HDL CHOLESTEROL	>40 (>50)		MG/DL	51.009(16.625)	50.845(16.035)	0.764
67.03	72.87	X956	9830-1	CHOLESTEROL:HDL RATIO	1	4.50 (<5.0)	RATIO	3.444(1.173)	3.342(1.116)	0.631
67.35	73.22	X599	2571-8	TRIGLYCERIDES	<150		MG/DL	120.268(61.561)	122.985(64.578)	0.581
67.52	73.11	X601	13457-7	LDL CHOLESTEROL CALCULATED	<100 (<130)		MG/DL	87.573(34.429)	83.182(34.584)	0.569
74.1	78.43	X603	13458-5	VLDL CALCULATED	5	40	MG/DL	24.308(12.754)	25.067(13.391)	0.61

A. 2lpan before the index date (GNSIS)

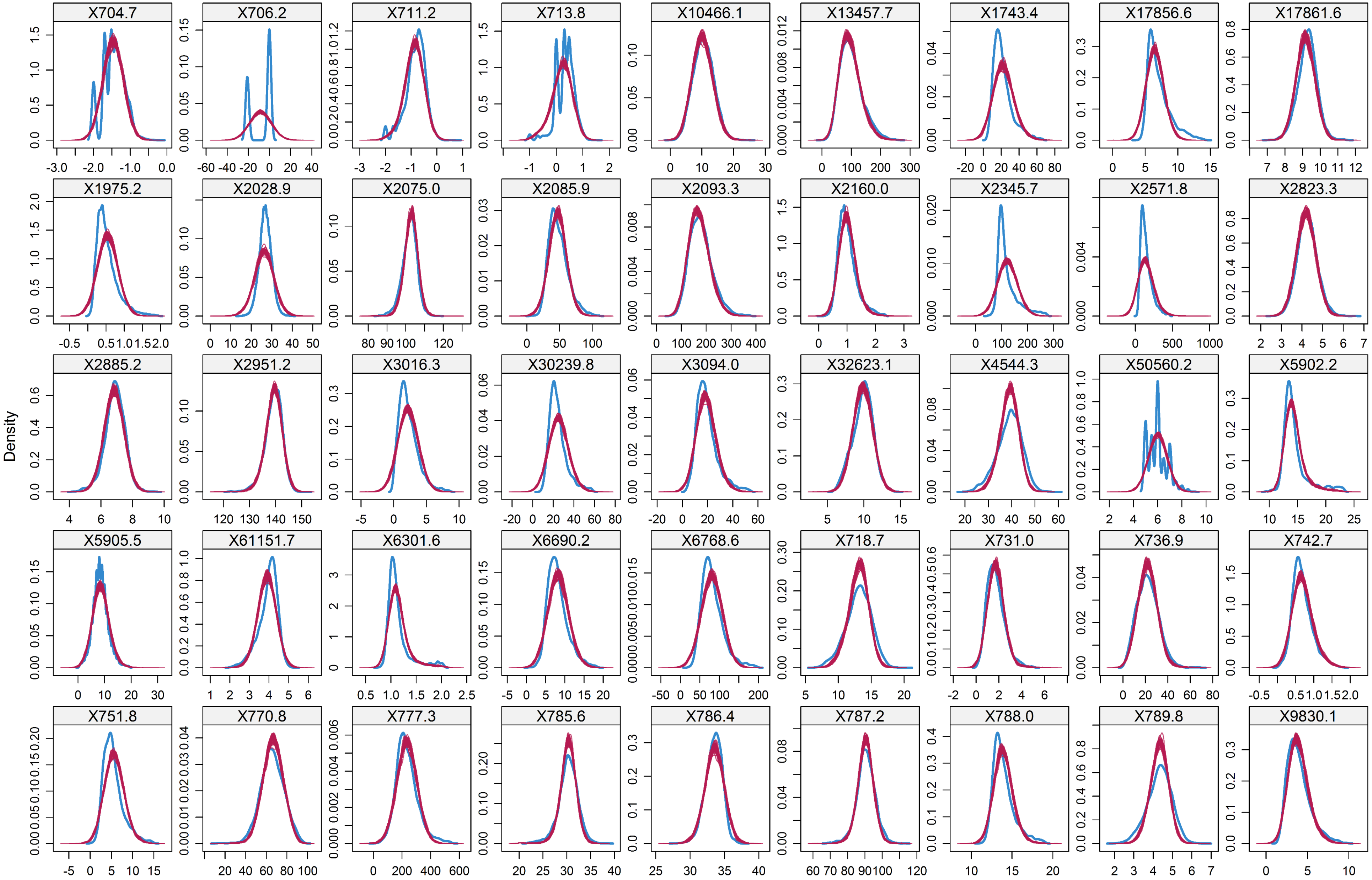

Supplementary Figure 1. Distribution of laboratory variables before (blue) and after (red) 50x MI of all missing values before the event in GNSIS (A) or HF (B) datasets using 2LPAN as an example.

B. 2lpan before the index date (HF)

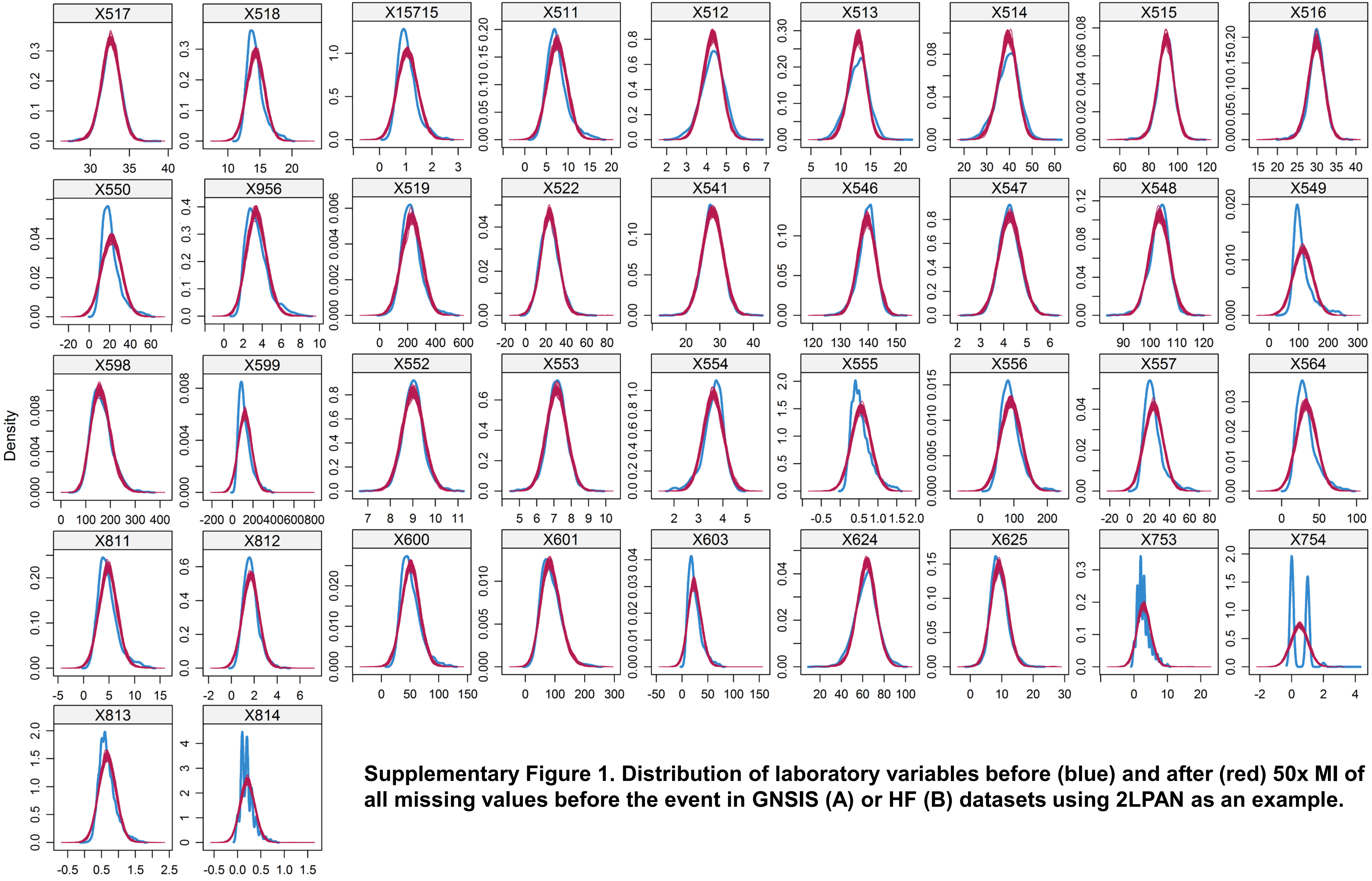

Supplementary Figure 1. Distribution of laboratory variables before (blue) and after (red) 50x MI of all missing values before the event in GNSIS (A) or HF (B) datasets using 2LPAN as an example.

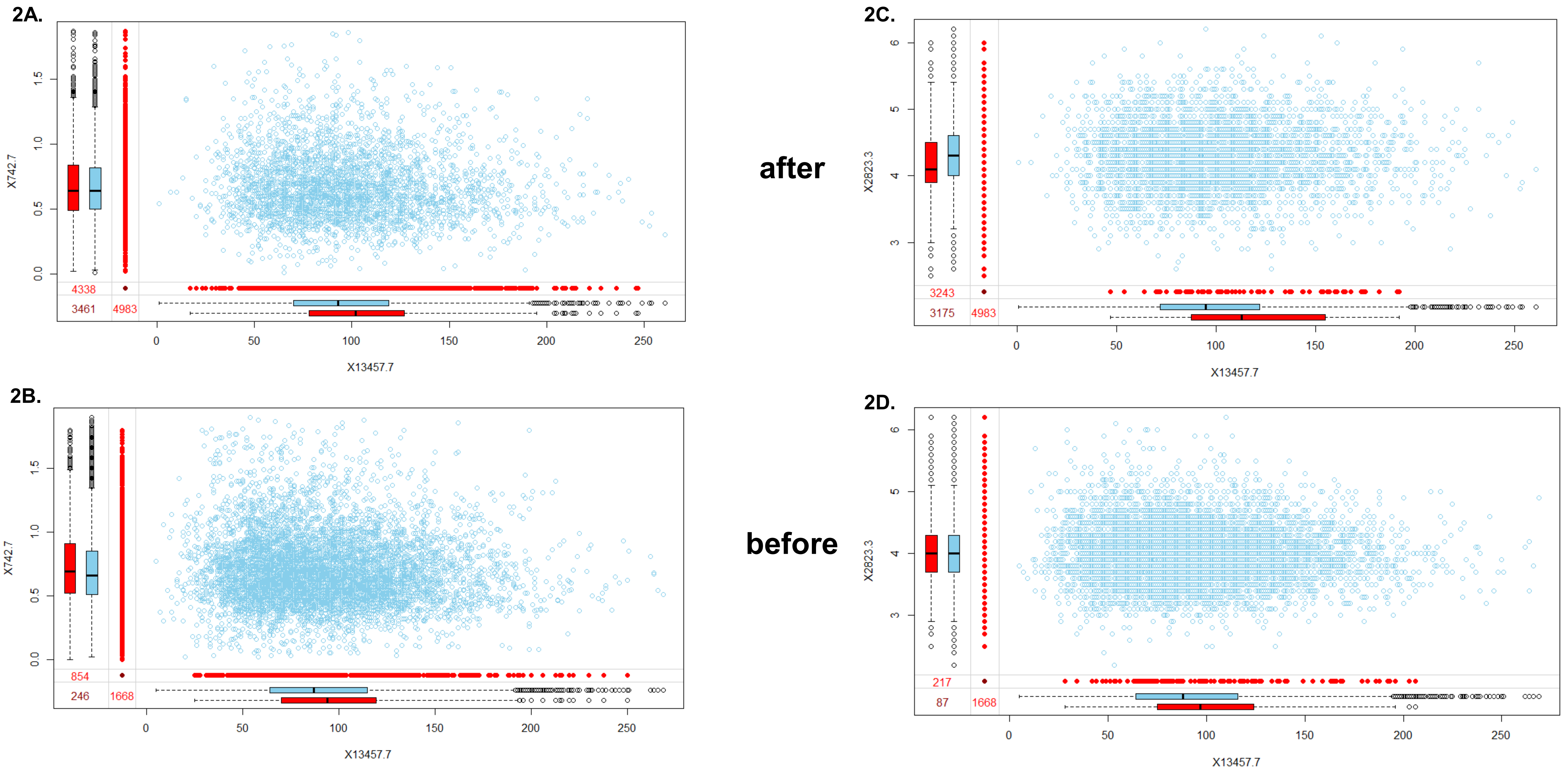

Supplementary Figure 2. Exploring missingness patterns and mechanisms. MCAR or MAR, between Lab 1 and Lab 2, before or after the event in GNSIS. All margin plots were created by the R "VIM" package. Lab 1 = cholesterol in LDL (13457-7); Lab 2 = Monocytes (742-7) or Potassium (2823-3) or Erythrocyte distribution width (788-0) or coagulation tissue induced factor (INR). Lab 2 was selected from one of each panel laboratory test other than the lipid panel where Lab 1 was collected from.

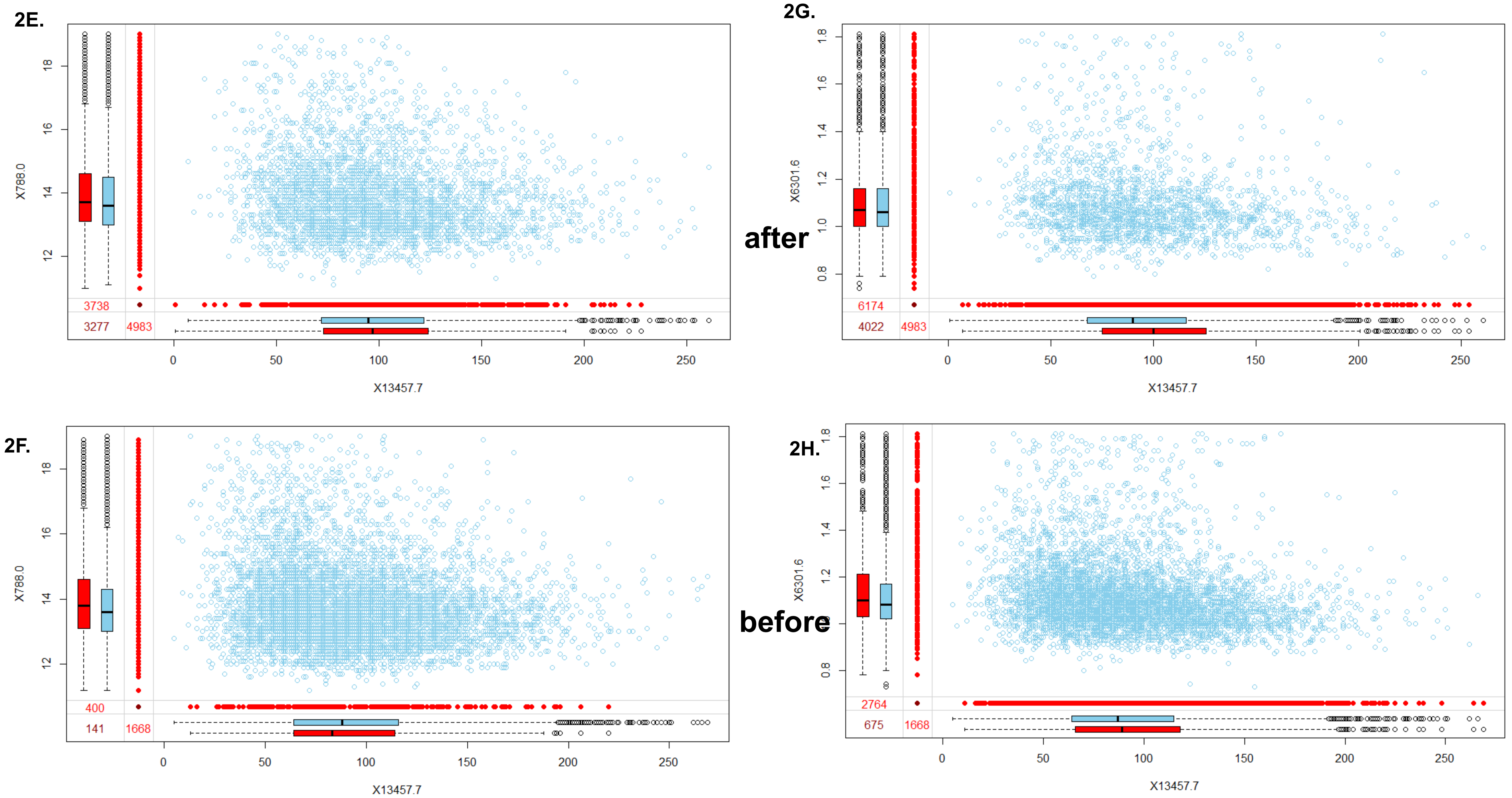

Supplementary Figure 2. Exploring missingness patterns and mechanisms. MCAR or MAR, between Lab 1 and Lab 2, before or after the event in GNSIS. All margin plots were created by the R "VIM" package. Lab 1 = cholesterol in LDL (13457-7); Lab 2 = Monocytes (742-7) or Potassium (2823-3) or Erythrocyte distribution width (788-0) or coagulation tissue induced factor (INR). Lab 2 was selected from one of each panel laboratory test other than the lipid panel where Lab 1 was collected from.

GNSIS

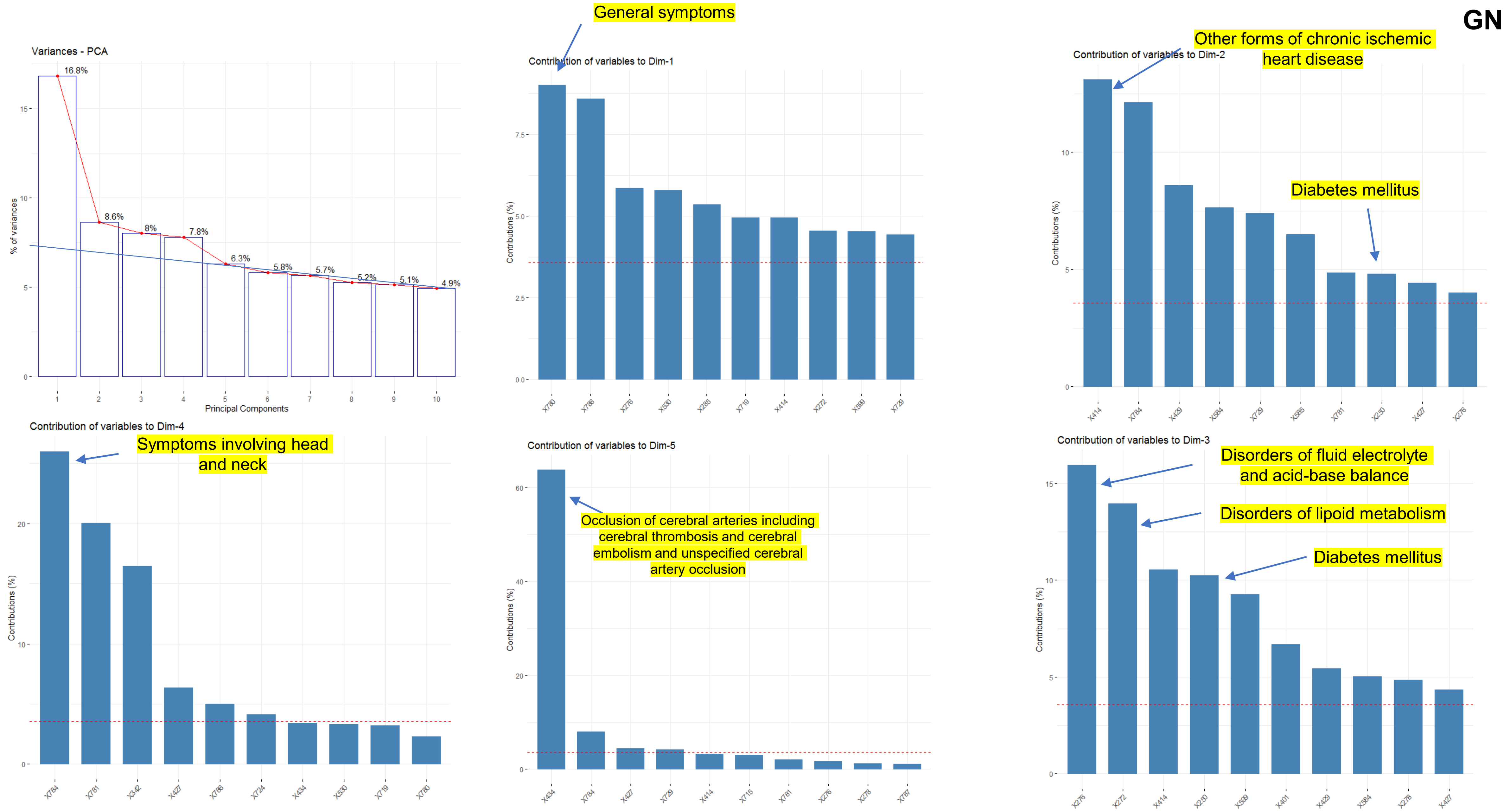

Supplementary Figure 3. **Principal Component Analysis on the comorbidity matrix of GNSIS (A) and HF (B).** The comorbidity matrix for ischemic stroke patients from the GNSIS database was created using ICD codes extracted from the Geisinger EHR database within an observation window of event ± 3years. A similar comorbidity matrix for HF cases was created using CCS codes extracted from the Sutter Health EHR database within an observation window of event ± 2 years. **A1/B1.** Scree plot; **A2-A6/B2-B6**. Top 10 ICD codes contribute to the major principal components, labeled as Dim, from Dim-1 to Dim-5. The straight line is intercepted with the red curved line at principal component = 5. Thus at least the main five principal components were selected as auxiliary variables.

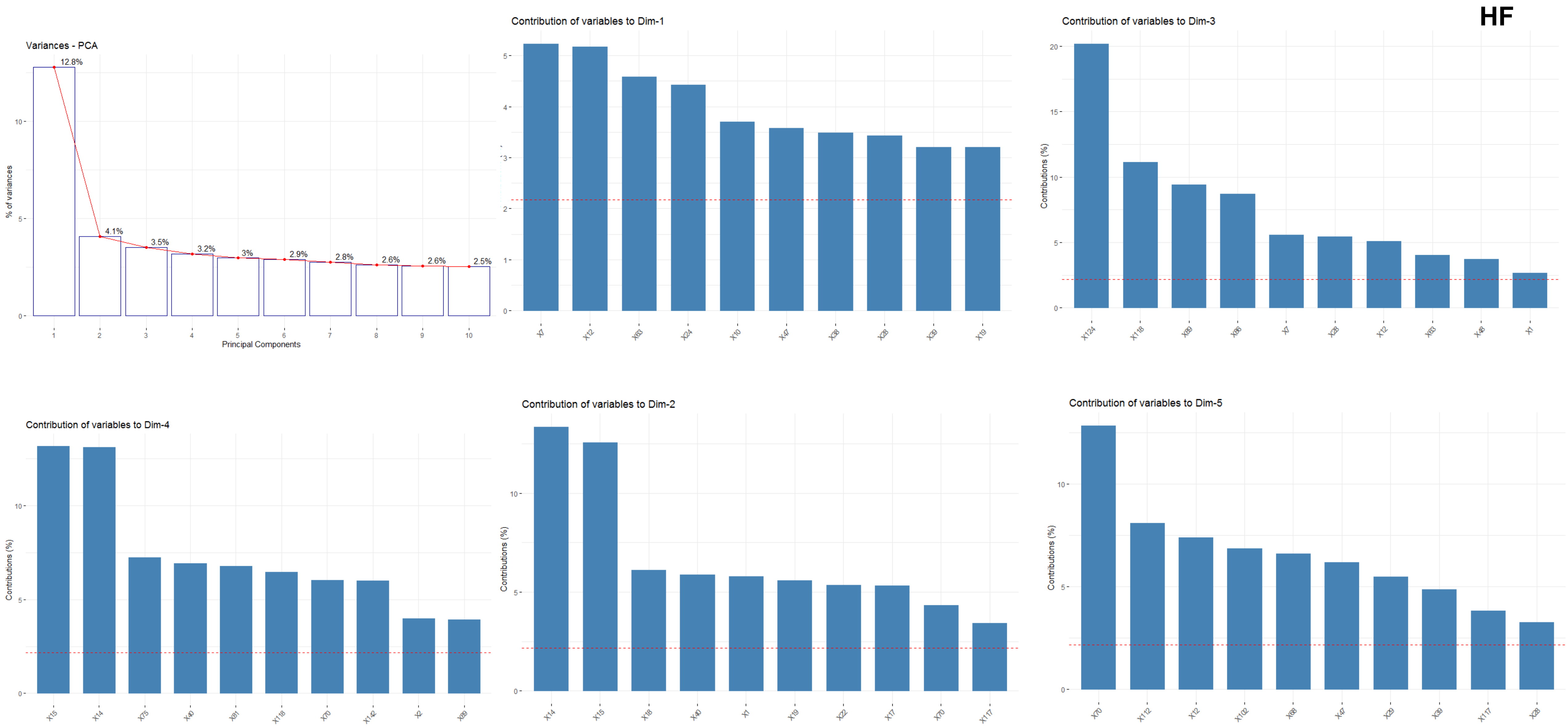

Supplementary Figure 3. **Principal Component Analysis on the comorbidity matrix of GNSIS (A) and HF (B).** The comorbidity matrix for ischemic stroke patients from the GNSIS database was created using ICD codes extracted from the Geisinger EHR database within an observation window of event ± 3years. A similar comorbidity matrix for HF cases was created using CCS codes extracted from the Sutter Health EHR database within an observation window of event ± 2 years. **A1/B1.** Scree plot; **A2-A6/B2-B6**. Top 10 ICD codes contribute to the major principal components, labeled as Dim, from Dim-1 to Dim-5. The straight line is intercepted with the red curved line at principal component = 5. Thus at least the main five principal components were selected as auxiliary variables.

Index	ccs_level3
X7	Other back problems
X12	Other non-traumatic joint disorders [204.]
X63	Genitourinary symptoms and ill-defined conditions [163.]
X24	Other nervous system symptoms and disorders
X10	Nonspecific chest pain [102.]
X47	Malaise and fatigue [252.]
X38	Abdominal pain [251.]
X28	Osteoarthritis [203.]
X39	Other and unspecified gastrointestinal disorders
X19	Esophageal disorders [138.]
X48	Other upper respiratory infections [126.]
X22	Other aftercare [257.]
X20	Other mycoses
X117	Urinary tract infections [159.]
X89	Deficiency and other anemia [59.]
X36	Other and unspecified lower respiratory disease
X60	Constipation
X122	Other ear and sense organ disorders [94.]
X75	Other malnutrition
X112	Disorders of the peripheral nervous system
X46	Conditions associated with dizziness or vertigo [93.]
X18	Depressive disorders [6572]
X45	Other and unspecified metabolic; nutritional; and endocrine disorders
X15	Other eye disorders [91.]
X1	Allergic reactions [253.]
X2	Other circulatory disease [117.]
X9	Cellulitis and abscess
X8	Medical examination/evaluation [256.]
X14	Cataract [86.]
X6	Other screening for suspected conditions (not mental disorders or infectious disease) [258.]
X124	Chronic kidney disease [158.]
X17	Other and unspecified benign neoplasm [47.]
X40	Blindness and vision defects [89.]
X68	Other thyroid disorders
X29	Obesity
X41	Administrative/social admission [255.]
X34	Congestive heart failure; nonhypertensive [108.]
X118	Coronary atherosclerosis and other heart disease [101.]
X86	Peripheral and visceral atherosclerosis [114.]
X70	Cardiac dysrhythmias [106.]
X84	Other chronic pulmonary disease
X4	Essential hypertension [98.]
X102	Heart valve disorders [96.]
X81	Conduction disorders [105.]
X93	Hyperplasia of prostate [164.]
X142	Peri-; endo-; and myocarditis; cardiomyopathy (except that caused by TB or STD) [97.]

Supplementary Figure 3. Description of CCS level 3 codes

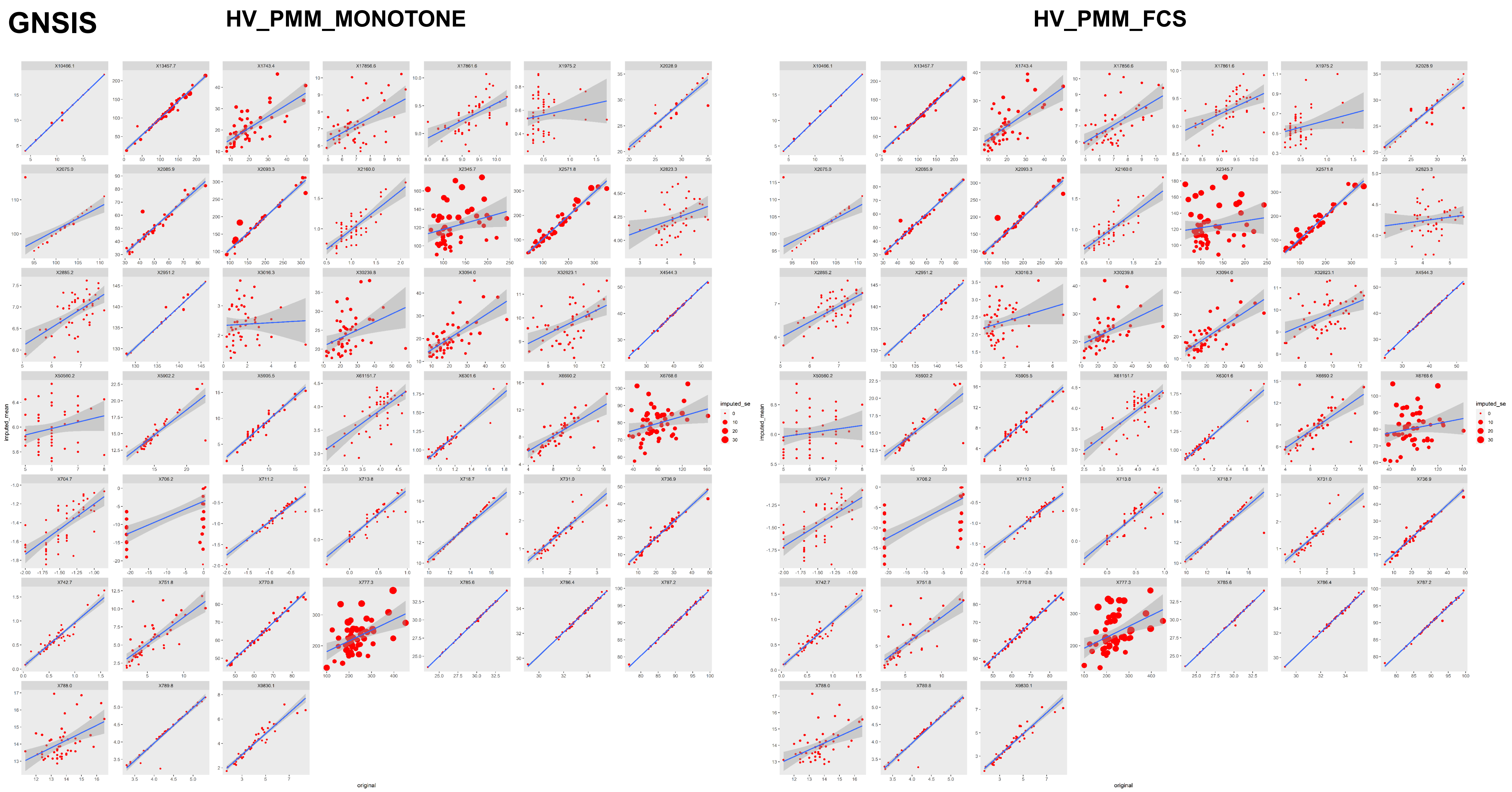

Supplementary Figure 4. Over-imputation plot for 50 holdout values (HV) or holdout complete cases (HC) in GNSIS or HF after 50 repeated multiple imputation.

GNSIS

HV_2LPAN_MONOTONE

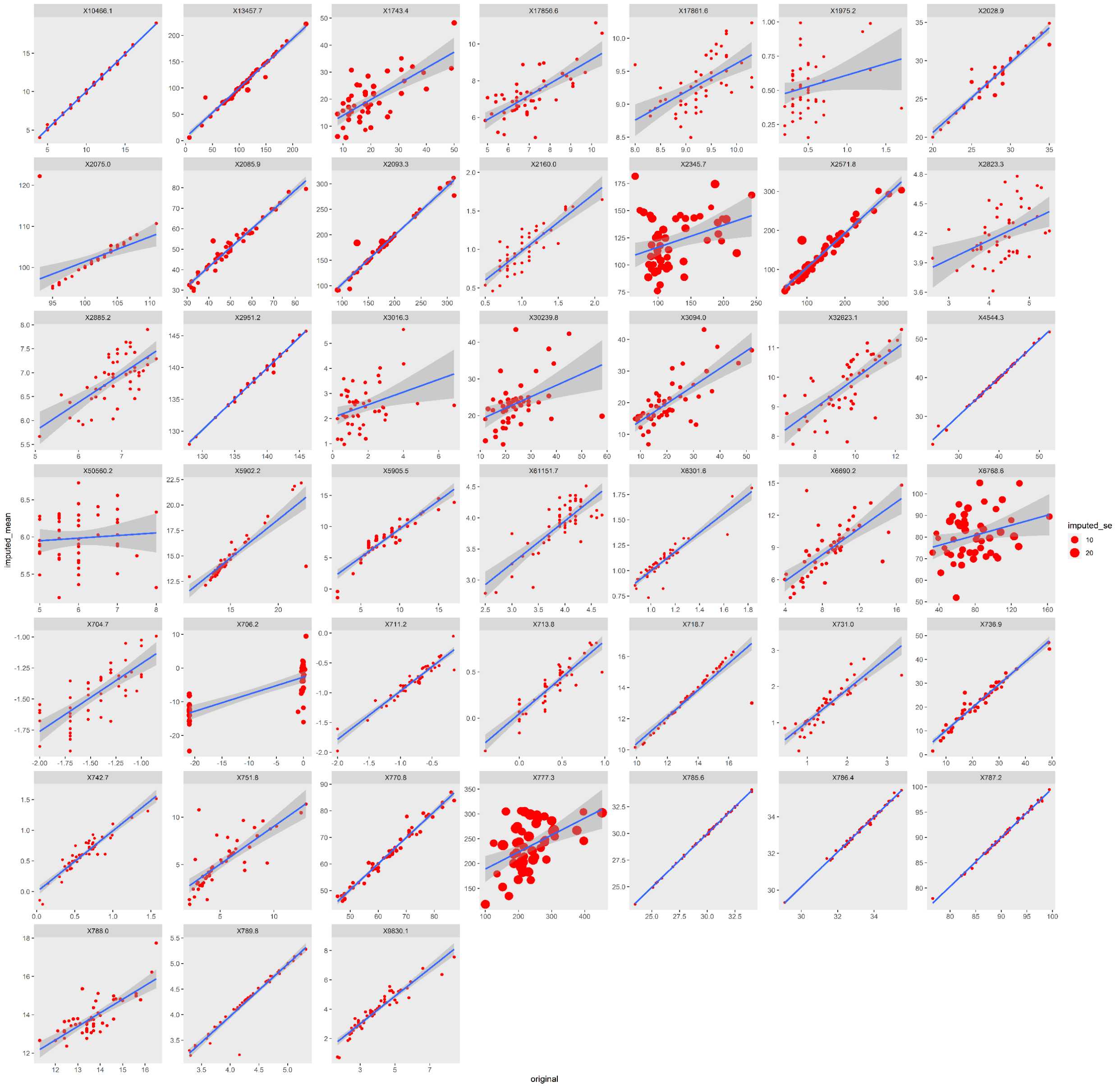

HV_2LPAN_FCS

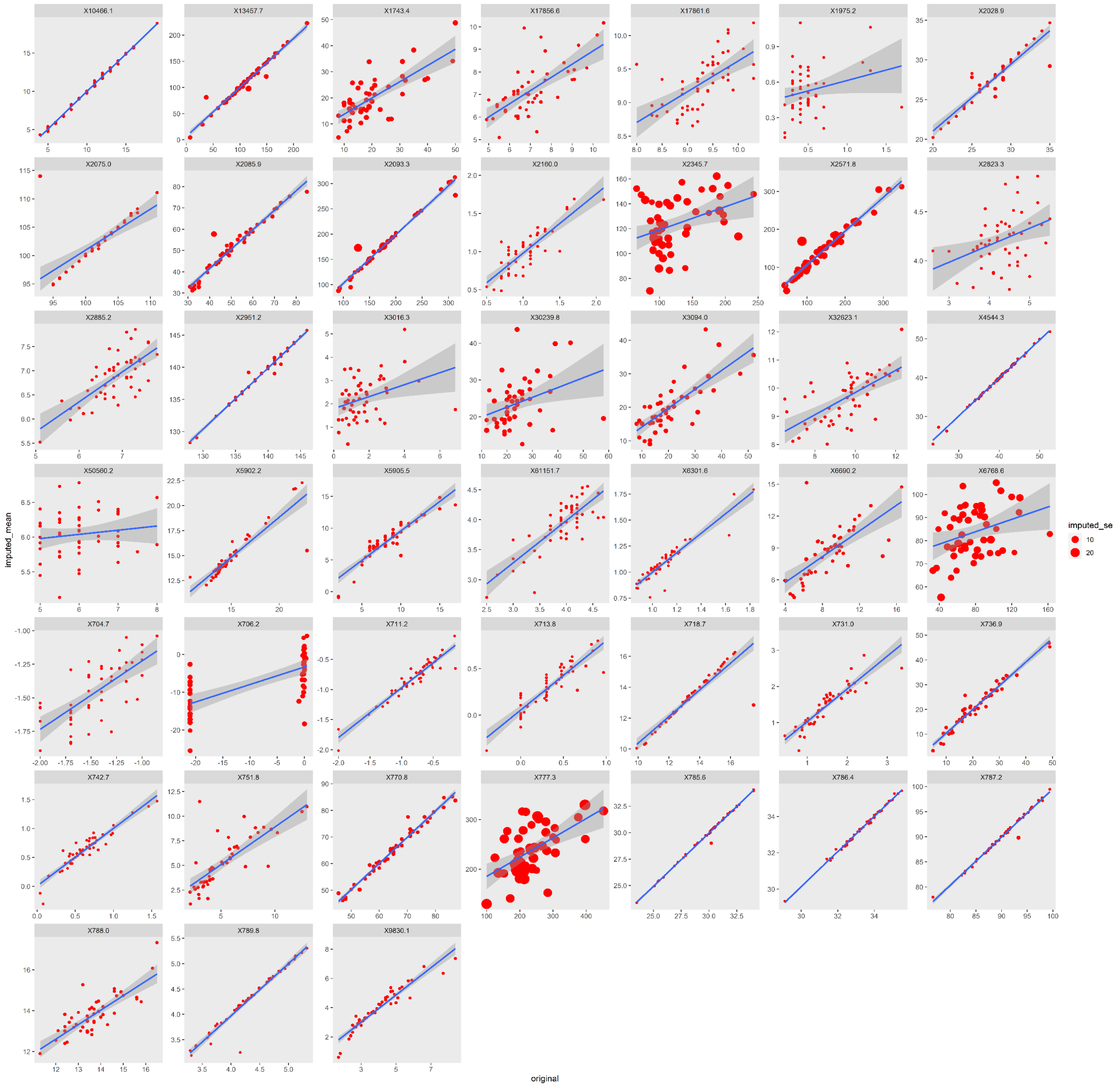

Supplementary Figure 4. Over-imputation plot for 50 holdout values (HV) or holdout complete cases (HC) in GNSIS or HF after 50 repeated multiple imputation.

GNSIS

HC_PMM_FCS

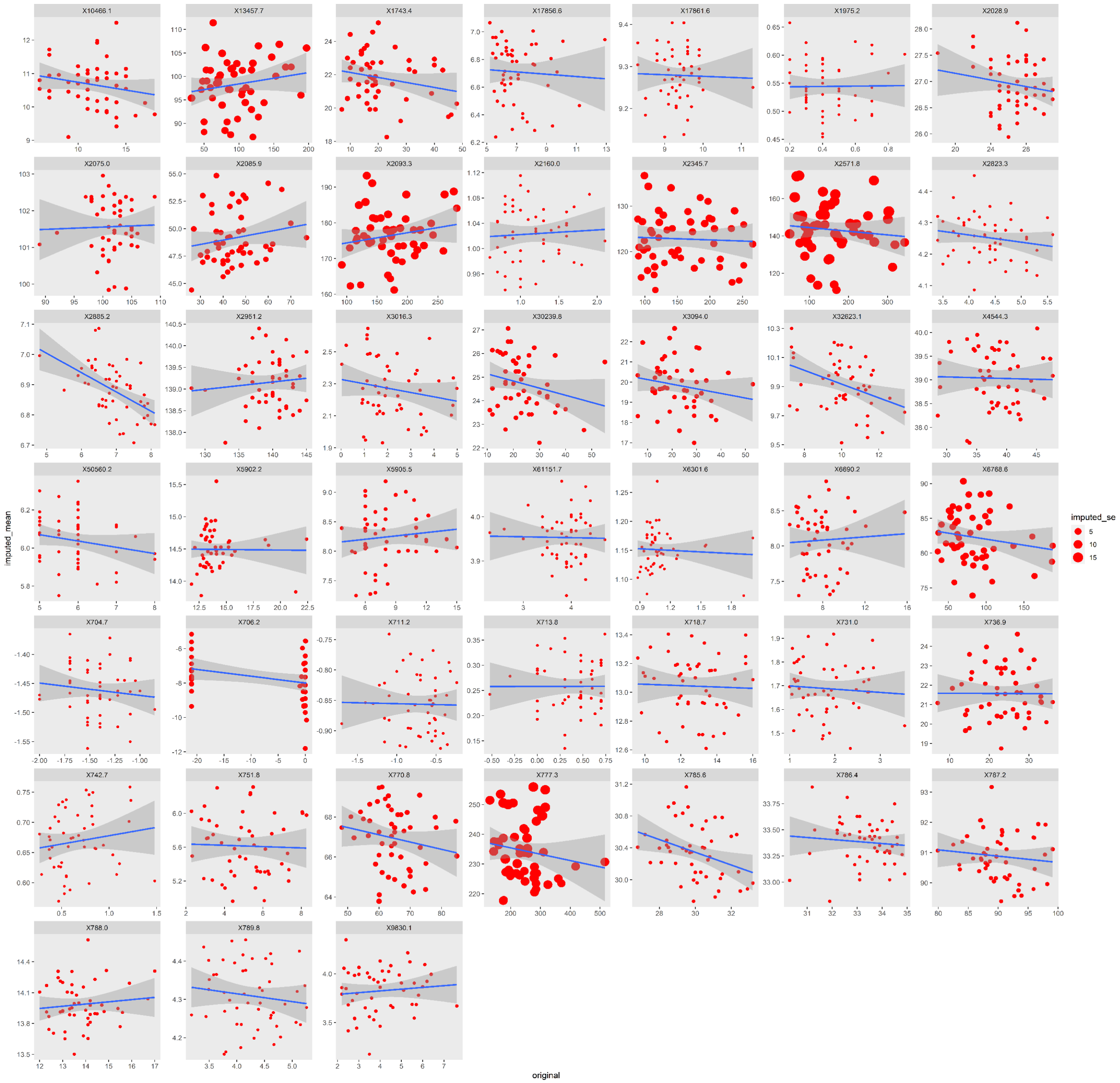

HC_PMM_MONOTONE

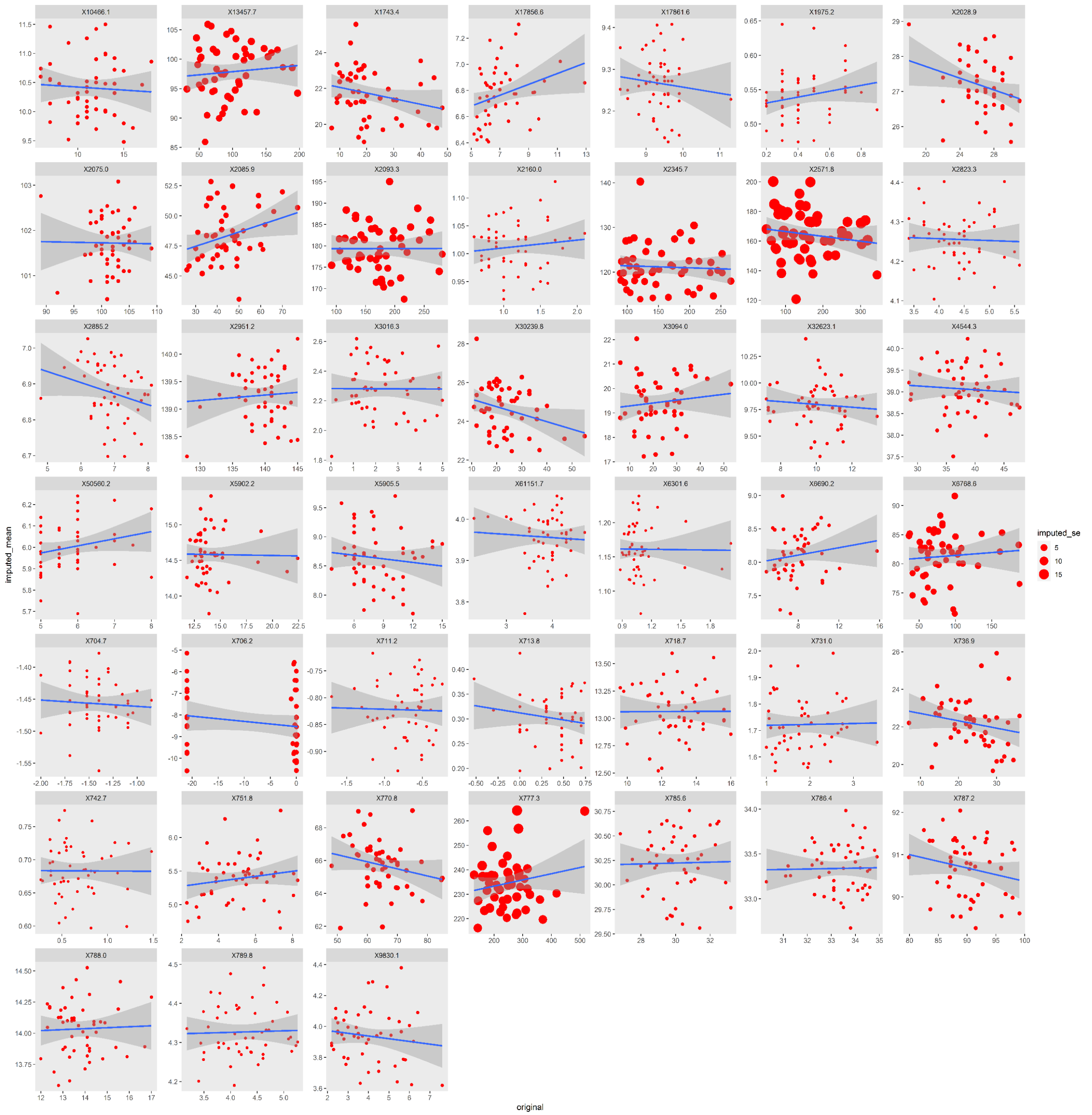

Supplementary Figure 4. Over-imputation plot for 50 holdout values (HV) or holdout complete cases (HC) in GNSIS or HF after 50 repeated multiple imputation.

GNSIS

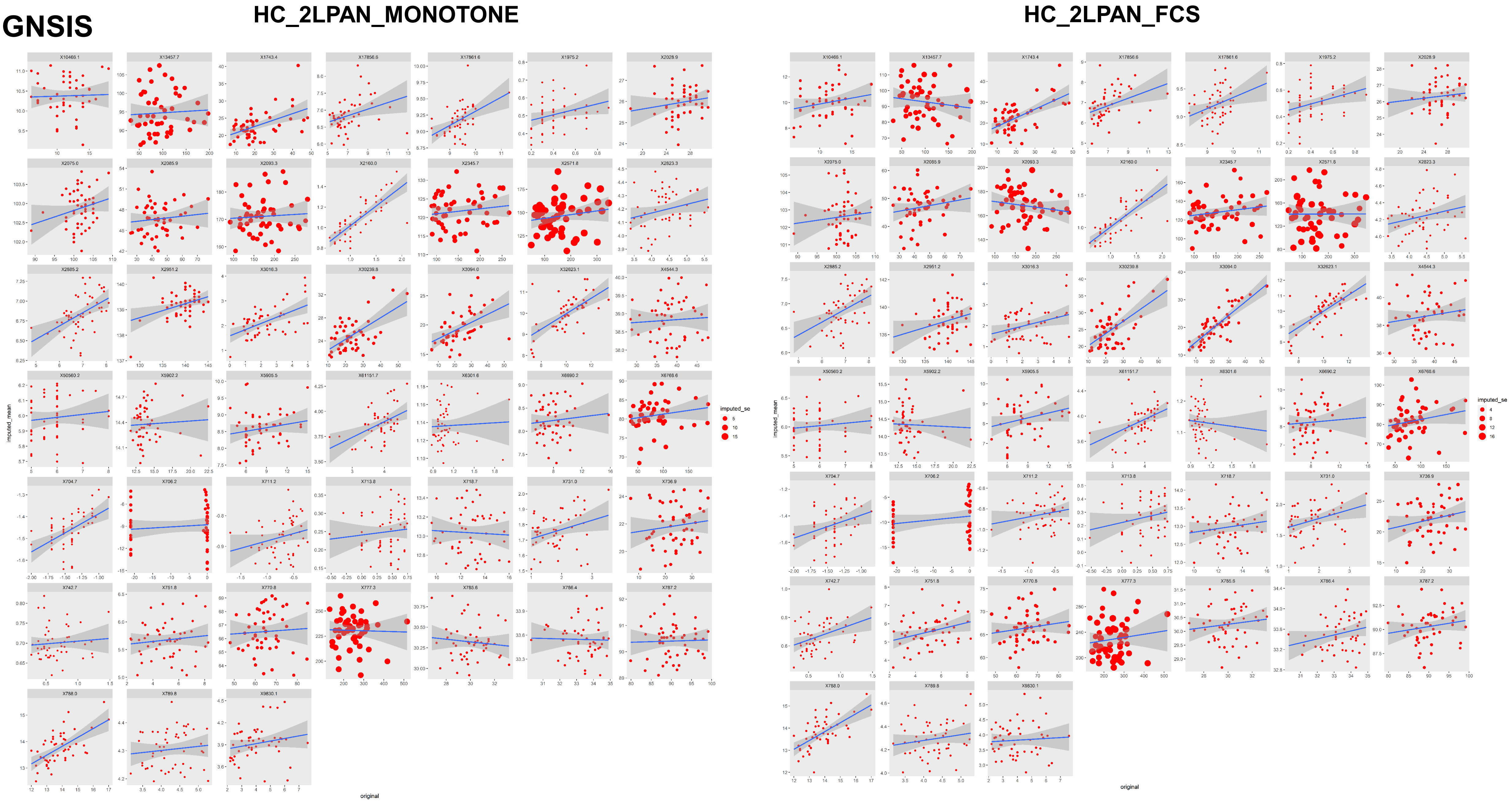

Supplementary Figure 4. Over-imputation plot for 50 holdout values (HV) or holdout complete cases (HC) in GNSIS or HF after 50 repeated multiple imputation.

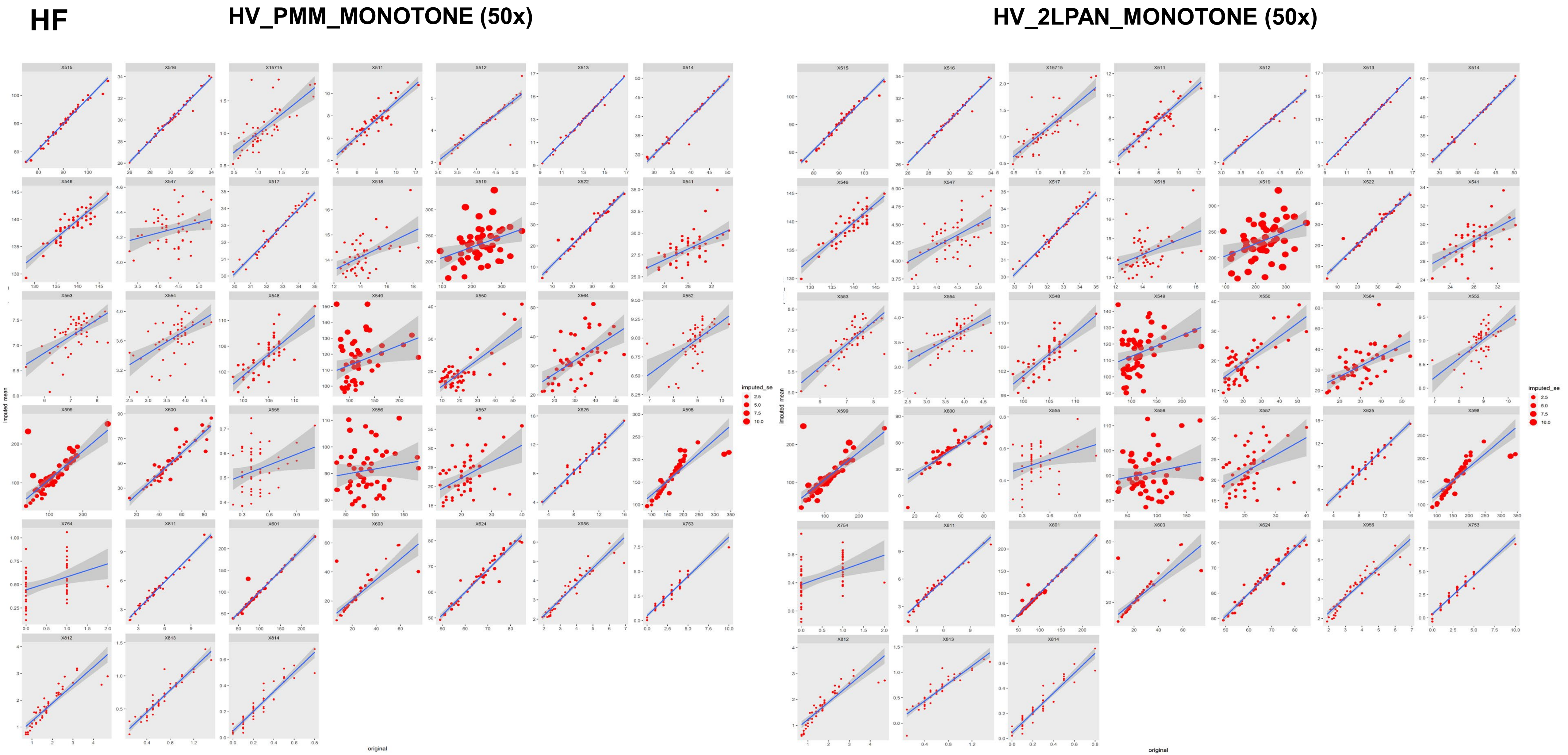

Supplementary Figure 4. Over-imputation plot for 50 holdout values (HV) or holdout complete cases (HC) in GNSIS or HF after 50 repeated multiple imputation.

HF

HV_PMM_FCS (50x)

HV_2LPAN_FCS (50x)

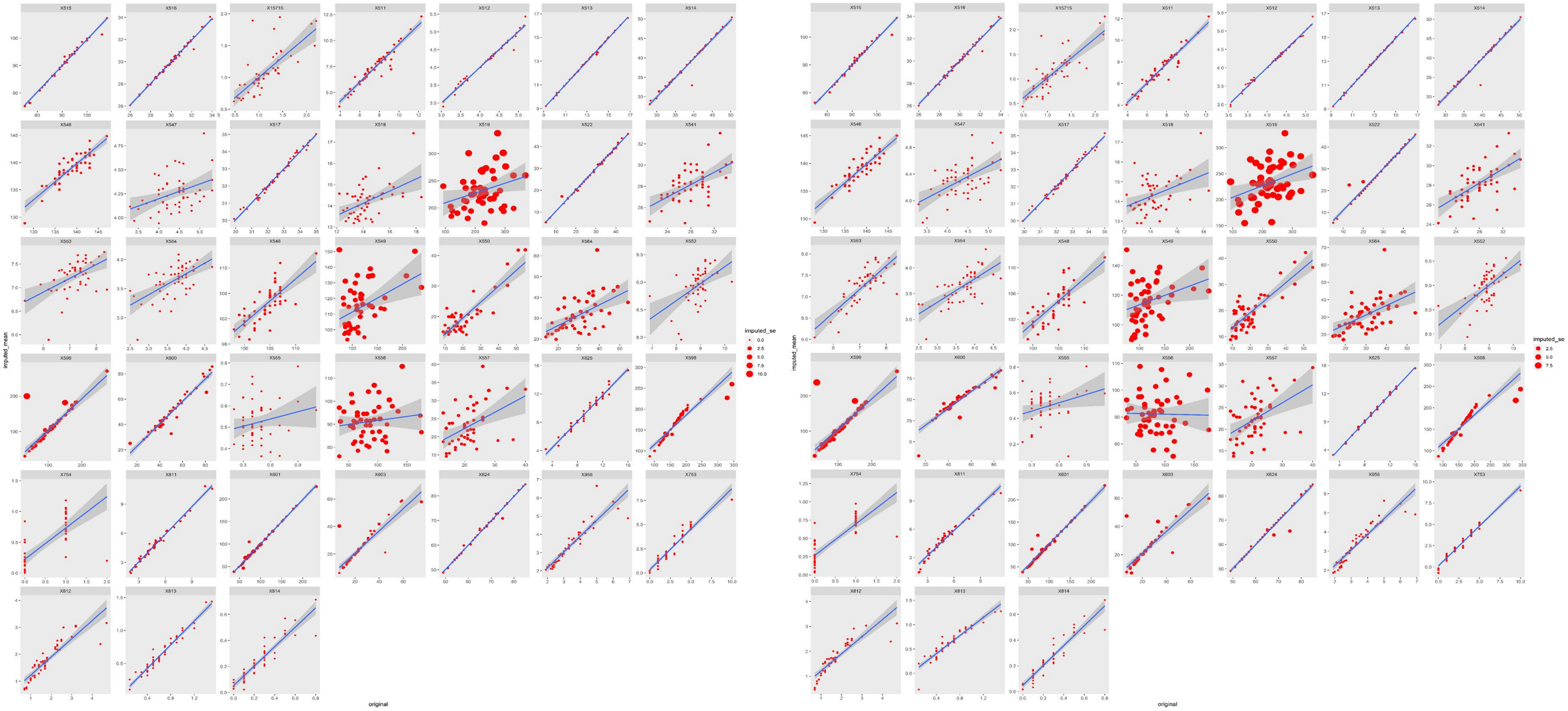

Supplementary Figure 4. Over-imputation plot for 50 holdout values (HV) or holdout complete cases (HC) in GNSIS or HF after 50 repeated multiple imputation.

HF

HC_PMM_MONOTONE (50x)

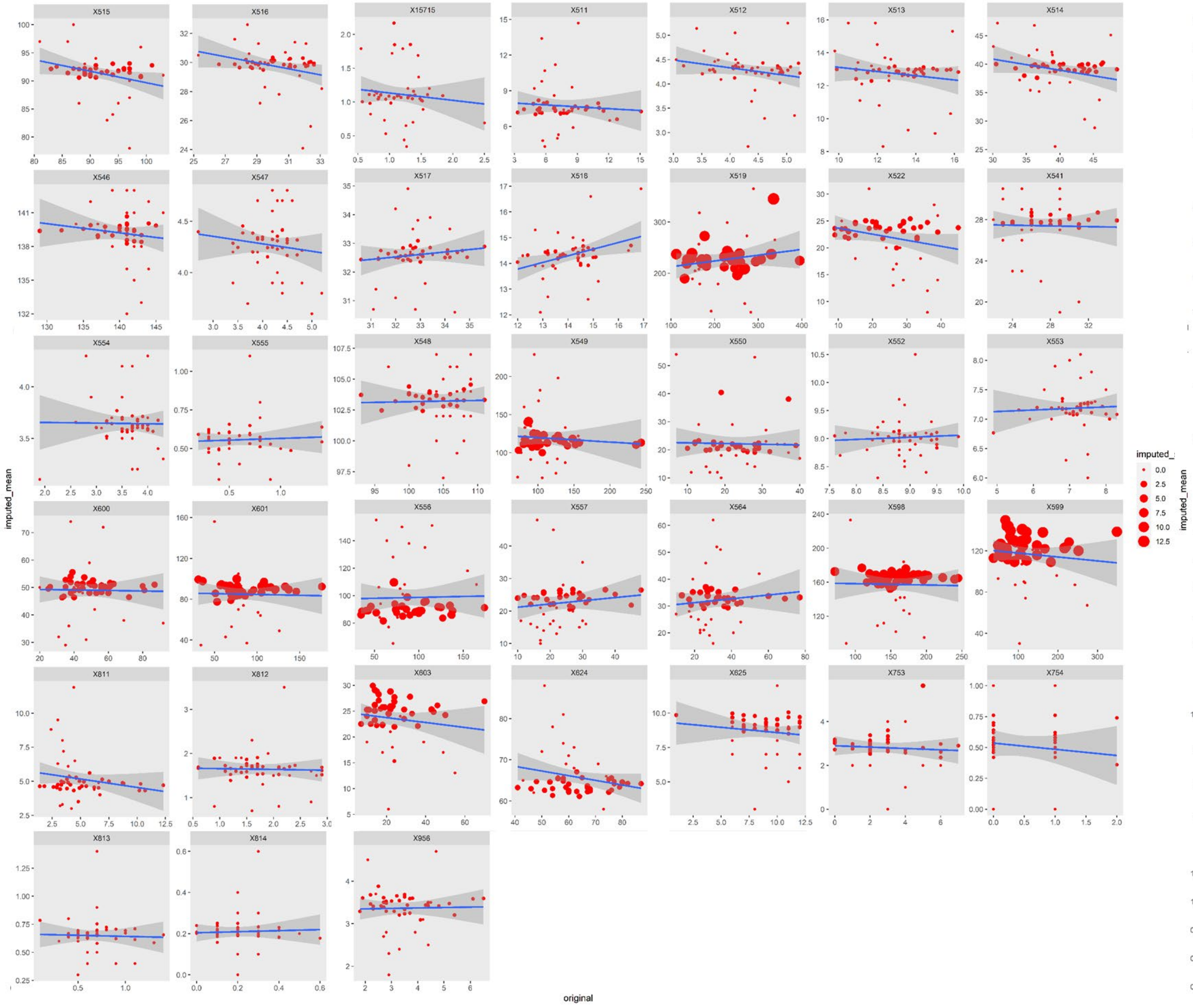

HC_PMM_FCS (50x)

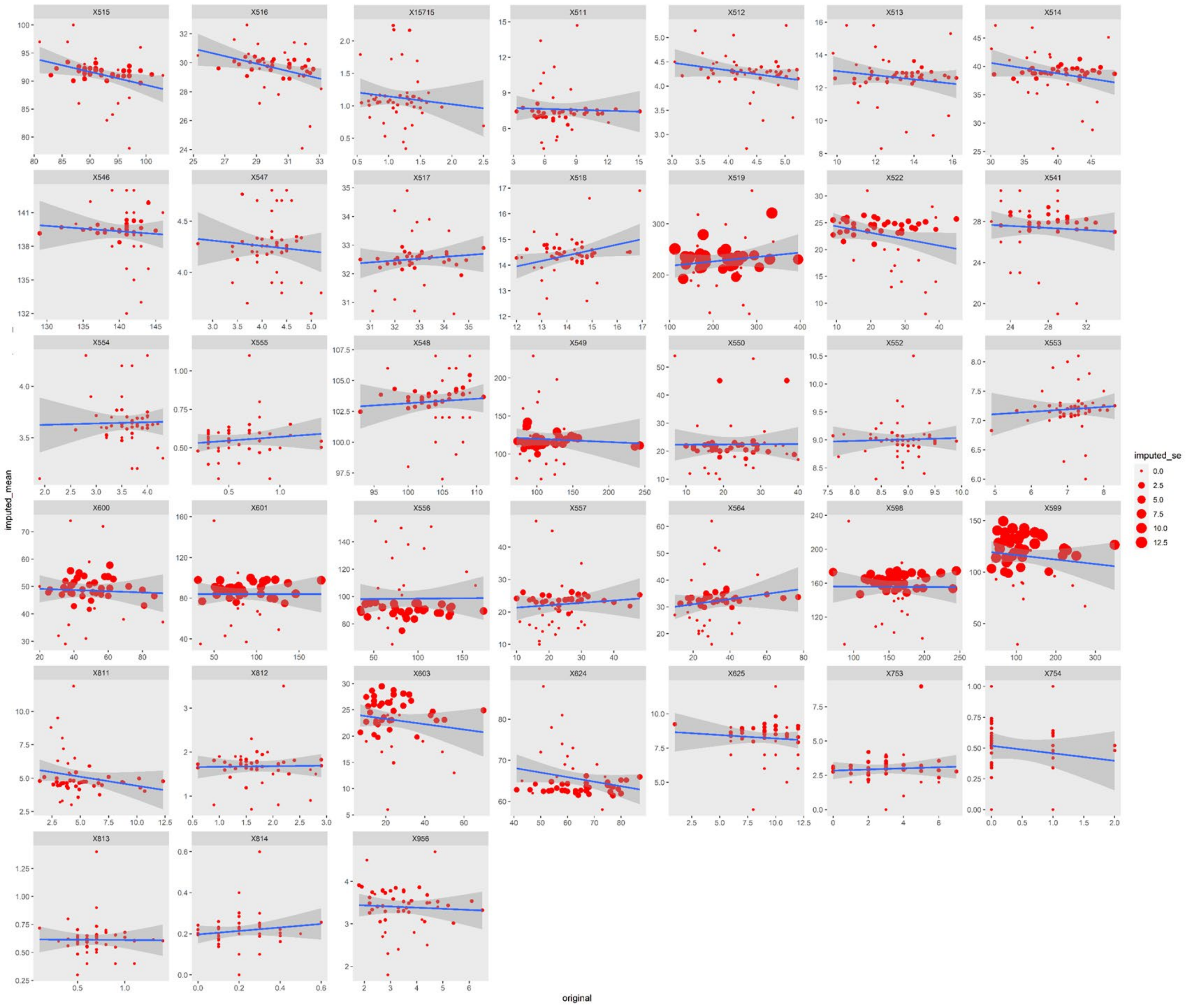

Supplementary Figure 4. Over-imputation plot for 50 holdout values (HV) or holdout complete cases (HC) in GNSIS or HF after 50 repeated multiple imputation.

HF

HC_2LPAN_MONOTONE (50x)

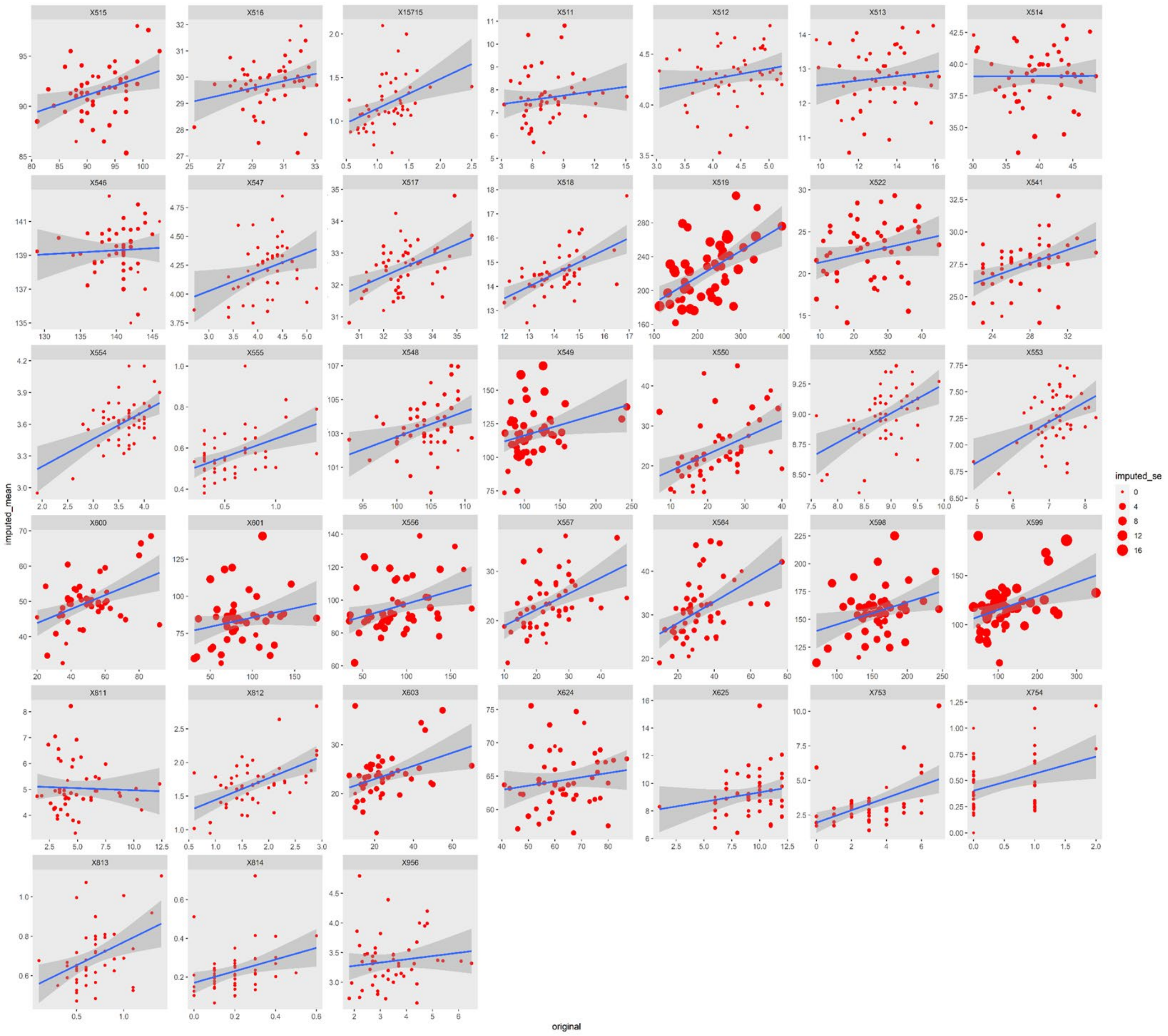

HC_2LPAN_FCS (50x)

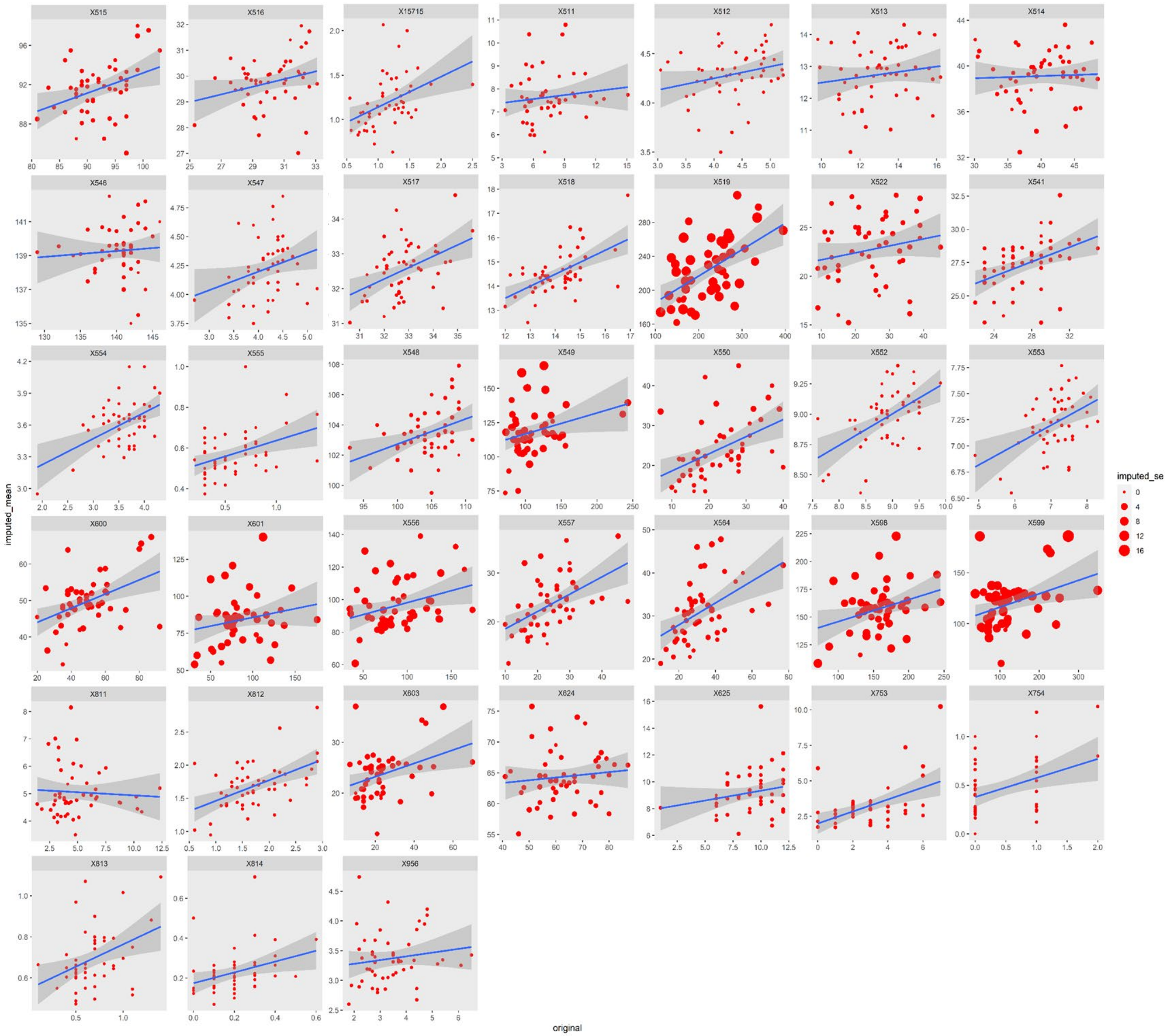

Supplementary Figure 4. Over-imputation plot for 50 holdout values (HV) or holdout complete cases (HC) in GNSIS or HF after 50 repeated multiple imputation.

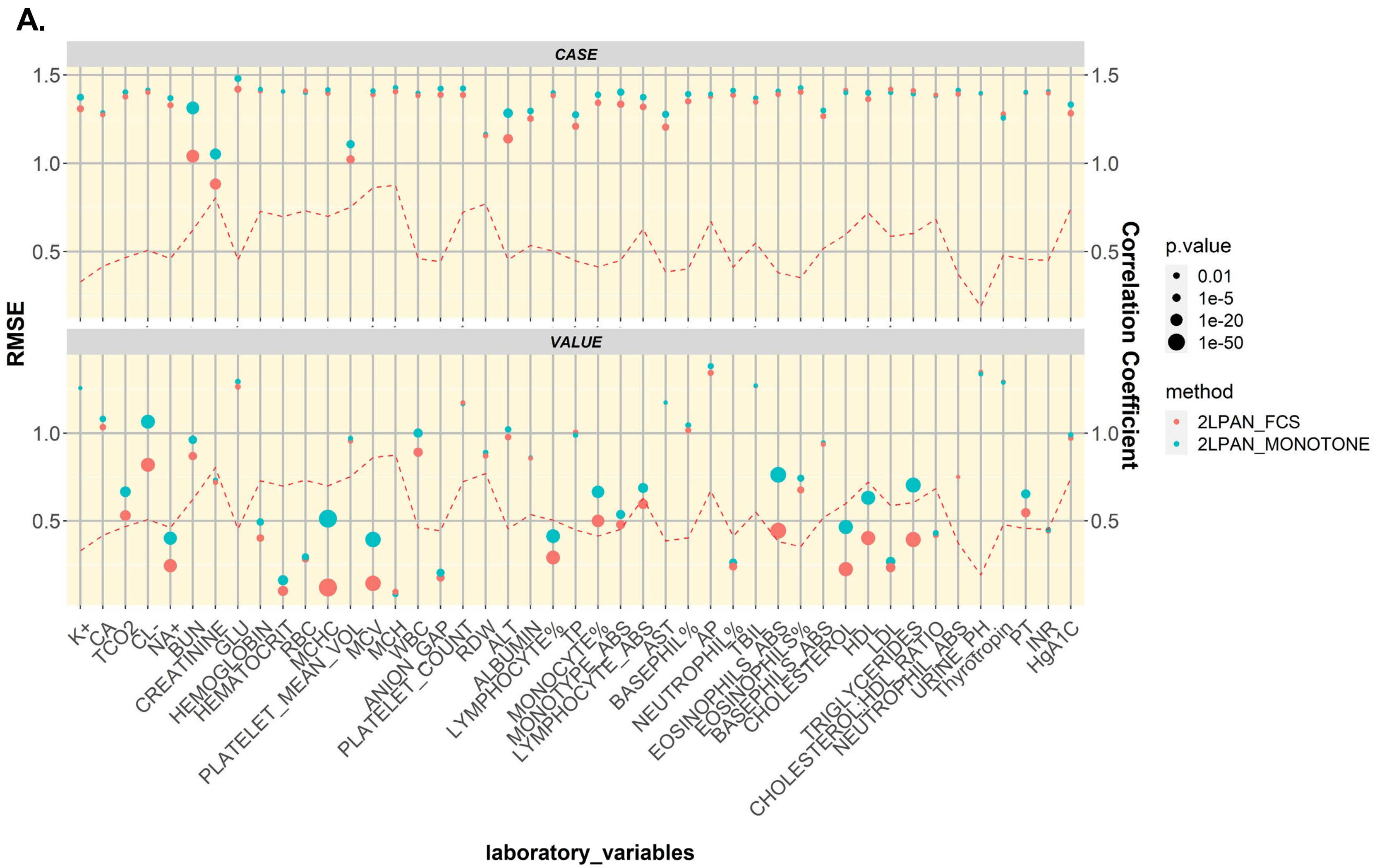

Supplementary Figure 5. Comparing the performance of 2LPAN with Markov Chain iteration (FCS) to 2LPAN without iteration (MONOTONE) after 50 repeated multiple imputation for random holdout cases (HC) or values (HV) using nRMSE in both GNSIS(A) and HF(B) datasets. Levene's test showed an equal variance of the nRMSE from two compared imputation algorithms, e.g. 2LPAN-FCS and PMM-FCS. Shapiro-Wilk test showed the normality of the difference for each comparison. An unpaired t-test was conducted to determine the mean difference of nRMSE between two compared imputation algorithms. Only the raw p-value < 0.05/45 (~0.0011) for GNSIS or < 0.05/38 (~0.0013) for HF was considered as statistical significance, which survived the Bonferroni correction for multiple testing. The curve for Pearson's correlation coefficient of observed values between before and after index date per variable was overlaid to the corresponding dot plot.

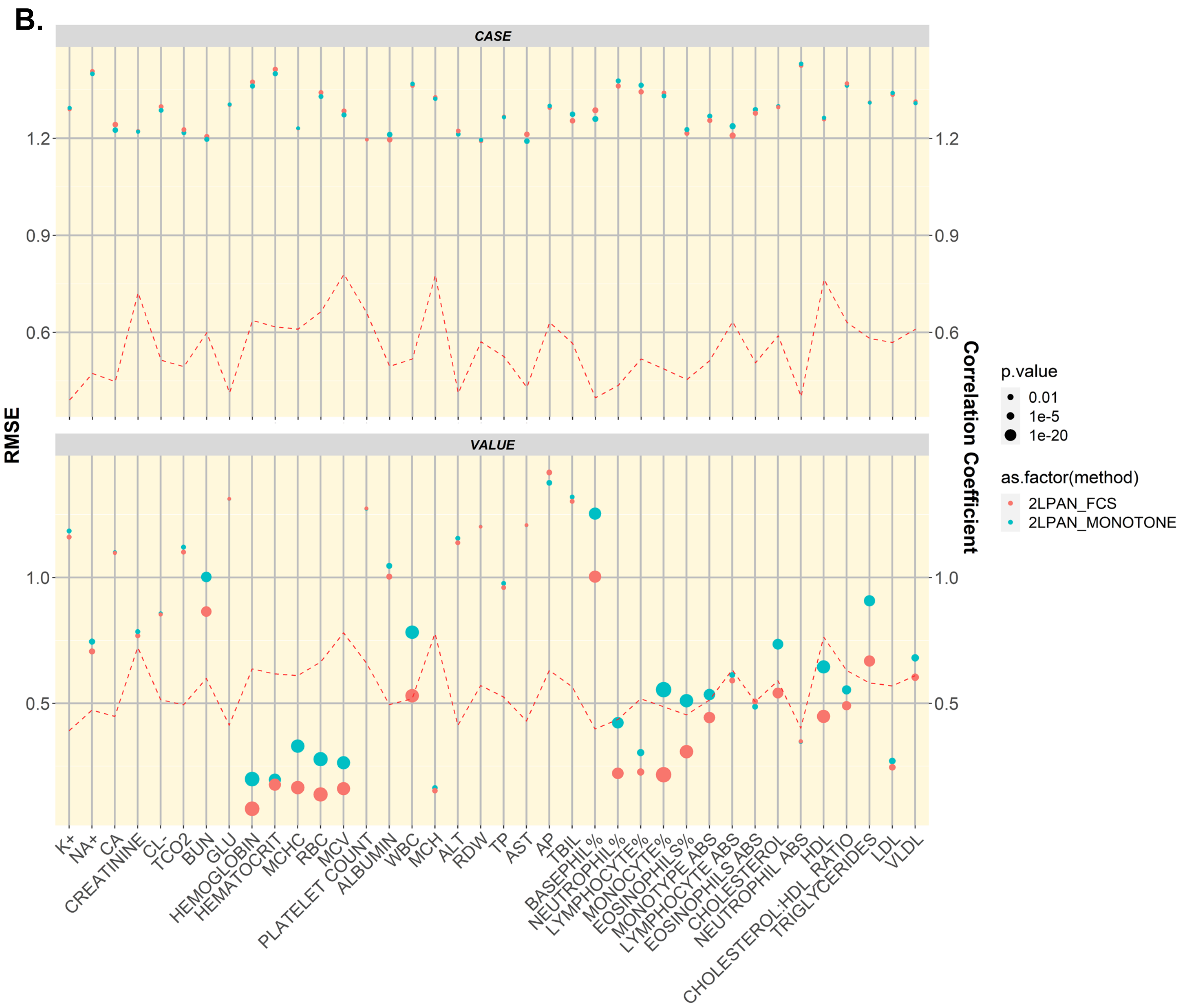

Supplementary Figure 5. Comparing the performance of 2LPAN with Markov Chain iteration (FCS) to 2LPAN without iteration (MONOTONE) after 50 repeated multiple imputation for random holdout cases (HC) or values (HV) using nRMSE in both GNSIS(A) and HF(B) datasets. Levene's test showed an equal variance of the nRMSE from two compared imputation algorithms, e.g. 2LPAN-FCS and PMM-FCS. Shapiro-Wilk test showed the normality of the difference for each comparison. An unpaired t-test was conducted to determine the mean difference of nRMSE between two compared imputation algorithms. Only the raw p-value < 0.05/45 (~0.0011) for GNSIS or < 0.05/38 (~0.0013) for HF was considered as statistical significance, which survived the Bonferroni correction for multiple testing. The curve for Pearson's correlation coefficient of observed values between before and after index date per variable was overlaid to the corresponding dot plot.

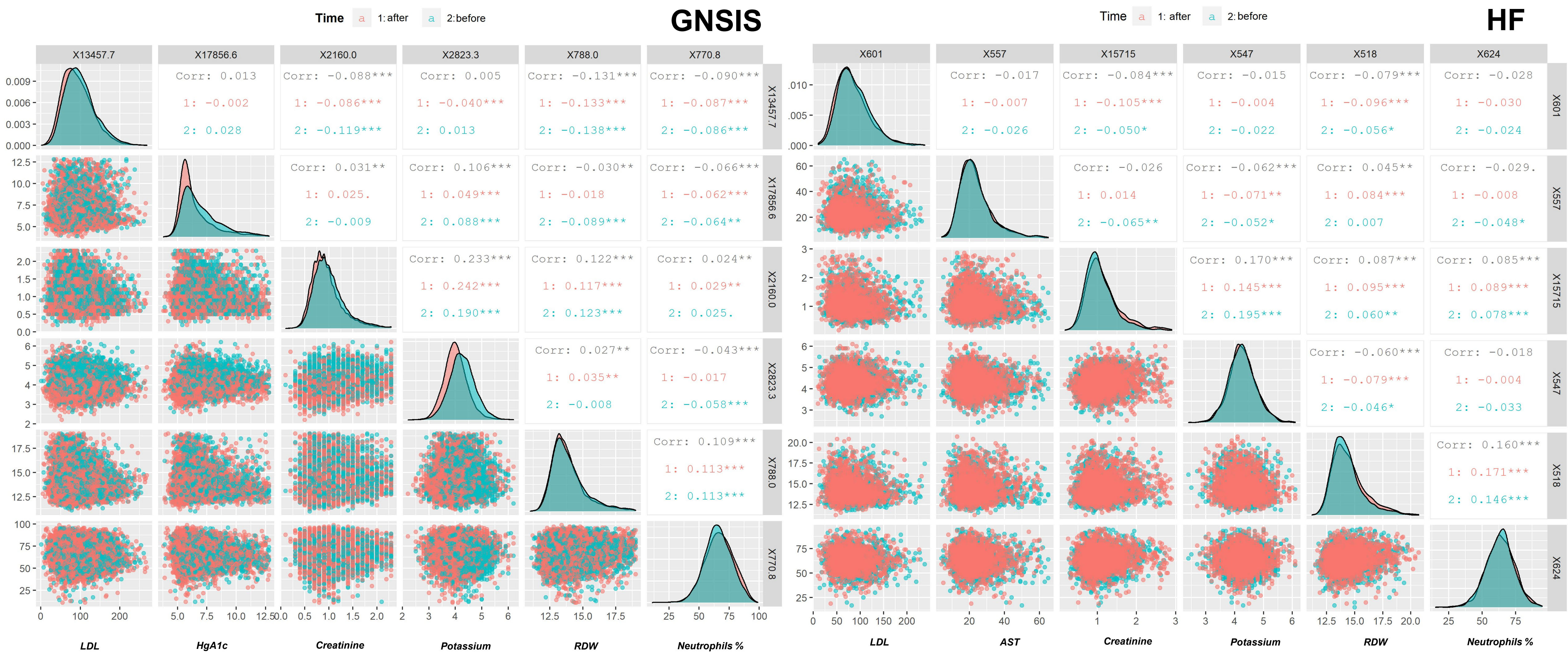

Supplementary Figure 6. Correlation matrix for selected laboratory variables from each panel test: GNSIS (A) and HF(B). Pearson's correlation coefficient between variables in the data before the event (blue), after the event (red), or both, were also listed. use = "pairwise.complete.obs"

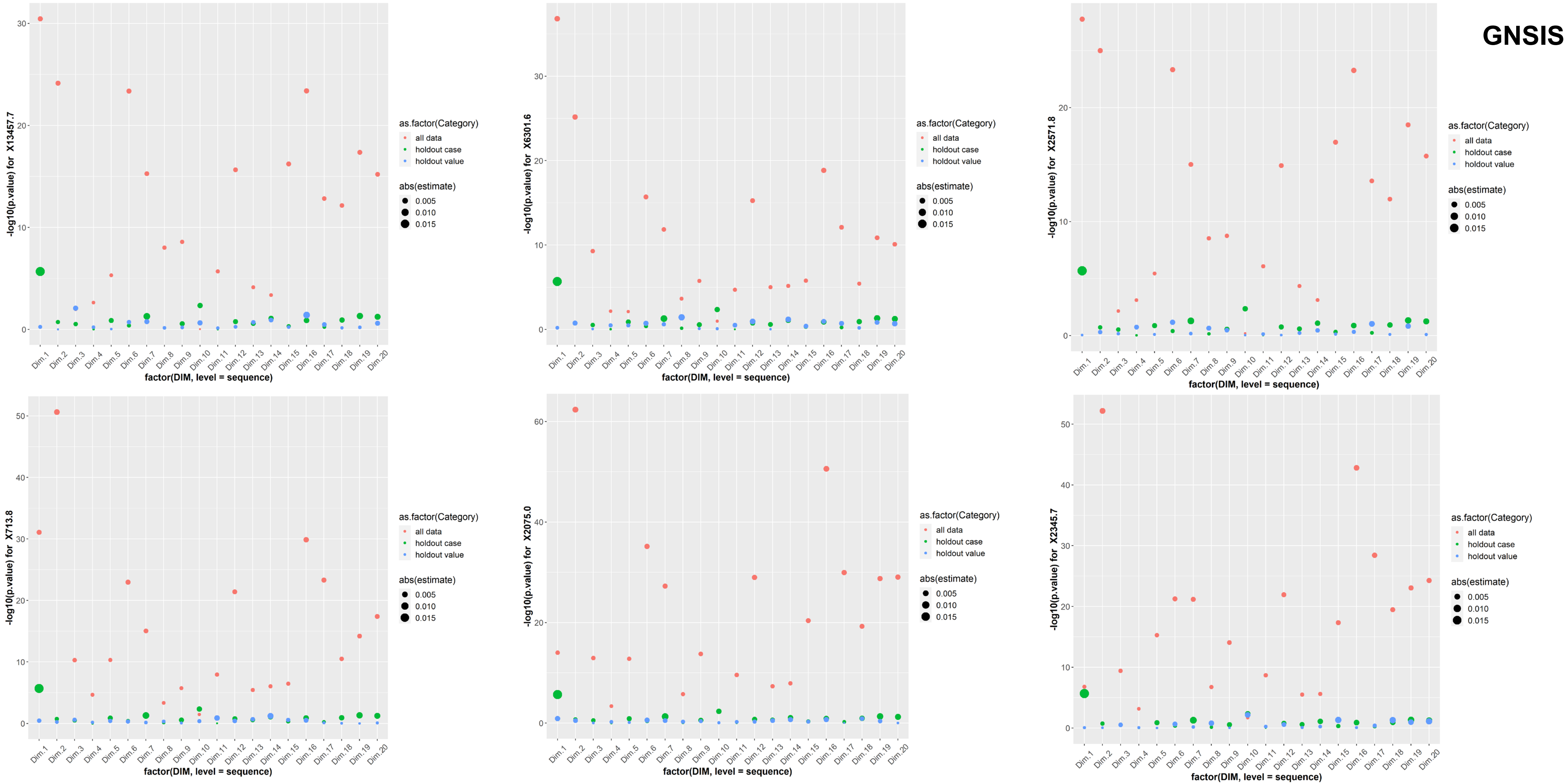

Supplementary Figure 7. This missingness (dummy variables) was highly associated with the comorbidity using the major 20 principal components (DIMs), as the latent variables in GNSIS (A) or HF dataset. The Y-axis represented log-transformed p-value of the significance after Welch unpaired t-test of the difference (absolute value) in DIM values between observed and missing groups. These six laboratory variables were randomly selected as examples for illustration. In both GNSIS and HF, the missingness was significantly associated with the majority of DIMs, particularly DIM.1. Compared to the observed entities, 50 cases with random holdout values (HV) had no significant difference in DIM values across all DIMs. In GNSIS, 50 cases with random holdout of complete cases (HC) have higher DIM values, particularly in DIM.1 whereas no significant difference in DIM values across all DIMs was observed in 50 HC compared to the corresponding observed entities.

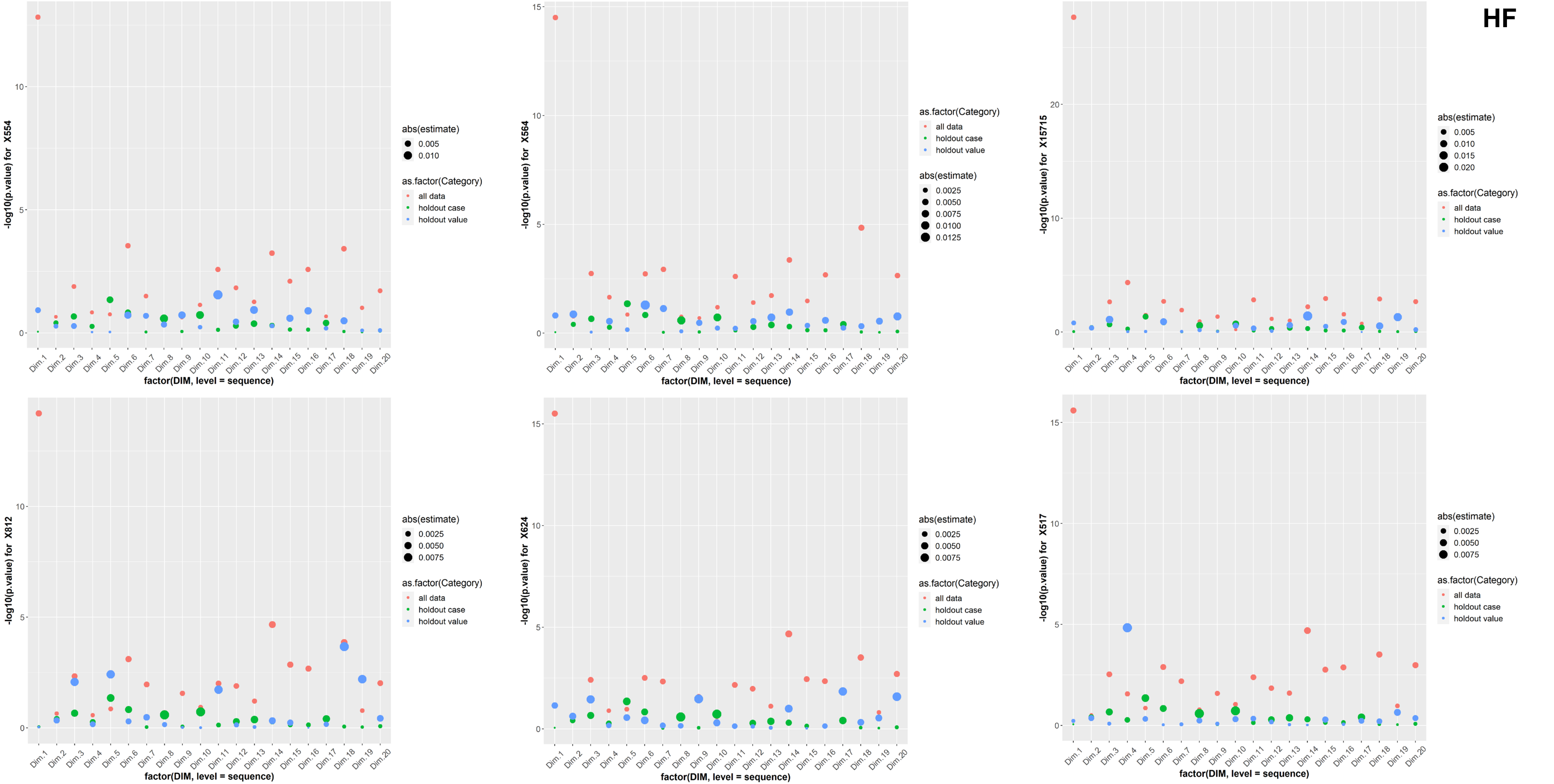

Supplementary Figure 7. This missingness (dummy variables) was highly associated with the comorbidity using the major 20 principal components (DIMs), as the latent variables in GNSIS (A) or HF dataset. The Y-axis represented log-transformed p-value of the significance after Welch unpaired t-test of the difference (absolute value) in DIM values between observed and missing groups. These six laboratory variables were randomly selected as examples for illustration. In both GNSIS and HF, the missingness was significantly associated with the majority of DIMs, particularly DIM.1. Compared to the observed entities, 50 cases with random holdout values (HV) had no significant difference in DIM values across all DIMs. In GNSIS, 50 cases with random holdout of complete cases (HC) have higher DIM values, particularly in DIM.1 whereas no significant difference in DIM values across all DIMs was observed in 50 HC compared to the corresponding observed entities.

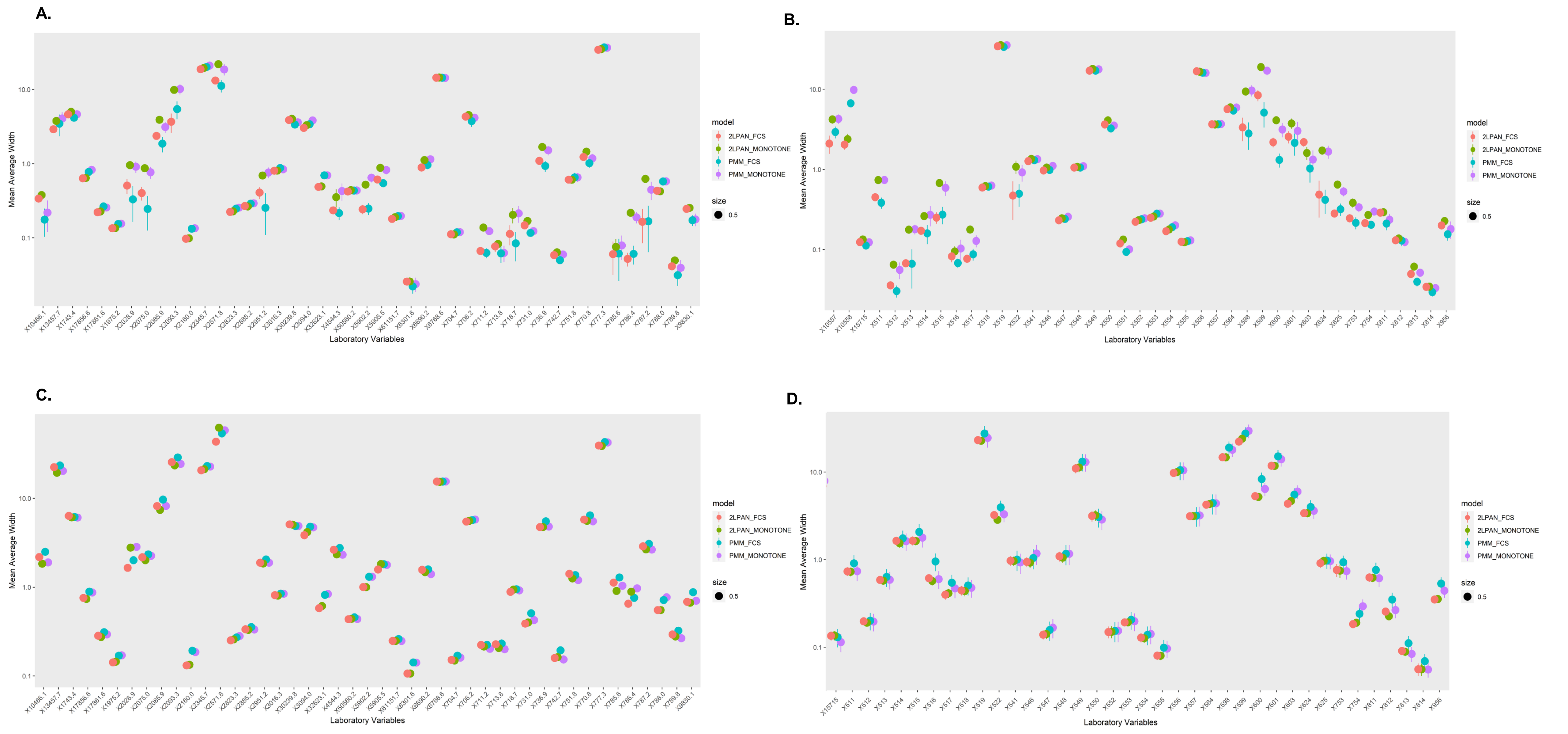

Supplementary Figure 8. Mean Average Width (AW), an indicator of statistical efficiency across all imputation algorithms evaluated for both GNSIS (A, B) and HF (C, D) datasets. AW represented the average width of the confidence intervals of 50 holdouts per laboratory variable. The vertical bar represents 95%CI of the corresponding mean AW for 50 complete sets. A and B represented AW for 50 holdout values (HV) in GNSIS and HF respectively; C and represents AW for 50 holdout cases (HC) in GNSIS and HF respectively.

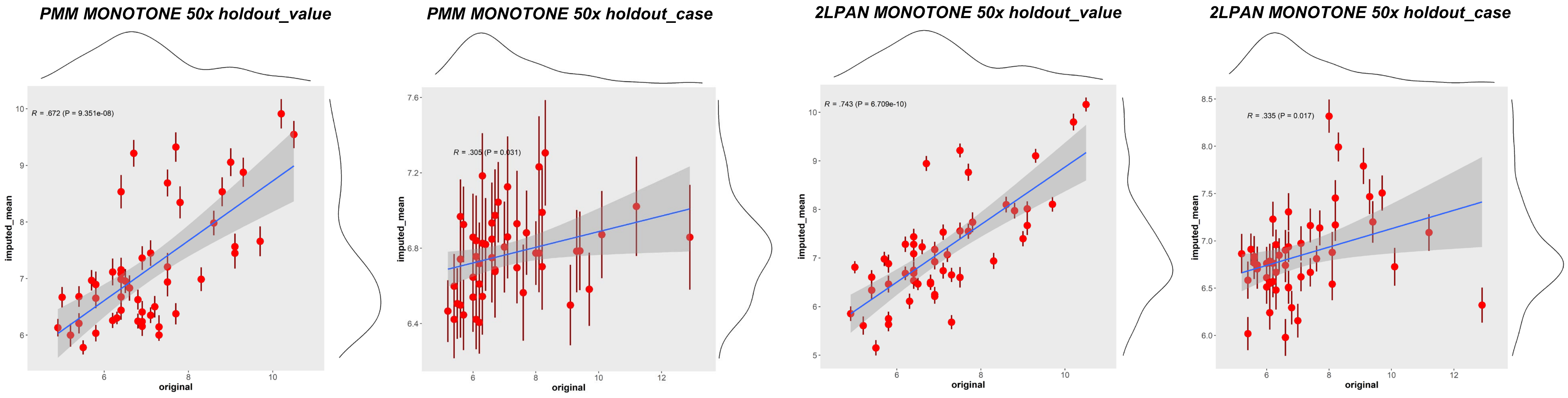

Supplementary Figure 9. Over-imputation plot demonstrated the mean and standard error of imputed values for the observed 50 holdouts where imputed values would lie if they were missing in the GNSIS dataset using HbA1c as an example. This figure was a supplement to Figure 7 in the main text. We only showed the monotone imputation for multivariate missing using PMM or 2LPAN for HV or HC. The lm fit line with 95% CI was superimposed on the scatter plot. The Pearson's correlation coefficient (R), as well as the significance of this correlation between 50 holdouts and imputed mean values were also present. This R value represented the optimal correlation coefficient which could be reached by each MI algorithm under multivariate or univariate setting**.** The kernel density plots at the margin of the scatter plot represented the corresponding distribution of observed and imputed 50 holdouts.
