# Supplementary material for: Imputation of missing values for electronic health record laboratory data
Source: NPJ Digit Med. 2021 Oct 11;4:147. doi: 10.1038/s41746-021-00518-0 (PMC8505441; doi:10.1038/s41746-021-00518-0)
Supplement: Supplementary file 2 — Reporting Summary [file 41746_2021_518_MOESM2_ESM.pdf]

nature portfolio

Corresponding author(s): Vida Abedi

Last updated by author(s): 07/10/2021

Reporting Summary

Nature Portfolio wishes to improve the reproducibility of the work that we publish. This form provides structure for consistency and transparency in reporting. For further information on Nature Portfolio policies, see our Editorial Policies and the Editorial Policy Checklist.

Statistics

For all statistical analyses, confirm that the following items are present in the figure legend, table legend, main text, or Methods section.

n/a	Confirmed	
☐	☒	The exact sample size (*n*) for each experimental group/condition, given as a discrete number and unit of measurement
☐	☒	A statement on whether measurements were taken from distinct samples or whether the same sample was measured repeatedly
☐	☒	The statistical test(s) used AND whether they are one- or two-sided<br>*Only common tests should be described solely by name; describe more complex techniques in the Methods section.*
☐	☒	A description of all covariates tested
☐	☒	A description of any assumptions or corrections, such as tests of normality and adjustment for multiple comparisons
☐	☒	A full description of the statistical parameters including central tendency (e.g. means) or other basic estimates (e.g. regression coefficient) AND variation (e.g. standard deviation) or associated estimates of uncertainty (e.g. confidence intervals)
☐	☒	For null hypothesis testing, the test statistic (e.g. *F*, *t*, *r*) with confidence intervals, effect sizes, degrees of freedom and *P* value noted<br>*Give P values as exact values whenever suitable.*
☐	☒	For Bayesian analysis, information on the choice of priors and Markov chain Monte Carlo settings
☐	☒	For hierarchical and complex designs, identification of the appropriate level for tests and full reporting of outcomes
☐	☒	Estimates of effect sizes (e.g. Cohen's *d*, Pearson's *r*), indicating how they were calculated

Our web collection on statistics for biologists contains articles on many of the points above.

Software and code

Policy information about availability of computer code

Data collection	Deidentified EHR data was deposited in the corresponding SQL database for either Geisinger GNSIS or Sutter Health HF. Data cleaning and process was conducted by R software version 3.6.2 with the following packages: dplyr, tidyr
Data analysis	R software version 3.6.2 was used for statistical analyses and the following packages were used for data analysis and visualization: VIM, naniar, missMDA, Amelia, mice, miceadds, missForest, randomForest, Rmisc, factoextra, FactoMineR, lattice, GGally, broom

For manuscripts utilizing custom algorithms or software that are central to the research but not yet described in published literature, software must be made available to editors and reviewers. We strongly encourage code deposition in a community repository (e.g. GitHub). See the Nature Portfolio guidelines for submitting code & software for further information.

Data

Policy information about availability of data

All manuscripts must include a data availability statement. This statement should provide the following information, where applicable:

- Accession codes, unique identifiers, or web links for publicly available datasets
- A description of any restrictions on data availability
- For clinical datasets or third party data, please ensure that the statement adheres to our policy

Codes and additional meta-data, summary plots and information can be found at https://github.com/TheDecodeLab/Imputation-LaboratoryValues-EHR_v2.0

Field-specific reporting

Please select the one below that is the best fit for your research. If you are not sure, read the appropriate sections before making your selection.

☒ Life sciences ☐ Behavioural & social sciences ☐ Ecological, evolutionary & environmental sciences

For a reference copy of the document with all sections, see nature.com/documents/nr-reporting-summary-flat.pdf

Life sciences study design

All studies must disclose on these points even when the disclosure is negative.

Sample size	Overall, 45 quantitative laboratory variables from GNSIS (n=9037) and 38 from HF (n=5192) with less than 75% missingness were analyzed in this study.
Data exclusions	Ordered and resulted laboratory tests completed within the index date ± 2 years for HF or index date ± 3 years for "Geisinger NeuroScience Ischemic Stroke (GNSIS)" were used for imputation, where the index date was defined as the first time the disease of interest (i.e. ischemic stroke or heart failure) meet the diagnosis criteria. Only quantitative laboratory values were considered for imputation.
Replication	Two distinct datasets were used: the GNSIS cohort and the Sutter Health heart failure cohort (HF), the latter of which was considered as replication cohort.
Randomization	We studied two missing patterns by randomly holding-out 50 laboratory values (HV) and 50 complete patient records (HC). To mimic Missing-completely-at-random (MCAR) or Missing-at-random (MAR) we used the HV and to mimic monotone missing we used the HC simulation.
Blinding	All investigators in this study had no control of missingness in EHR data collection.

Reporting for specific materials, systems and methods

We require information from authors about some types of materials, experimental systems and methods used in many studies. Here, indicate whether each material, system or method listed is relevant to your study. If you are not sure if a list item applies to your research, read the appropriate section before selecting a response.

Materials & experimental systems

n/a	Involved in the study
☒	☐ Antibodies
☒	☐ Eukaryotic cell lines
☒	☐ Palaeontology and archaeology
☒	☐ Animals and other organisms
☐	☒ Human research participants
☒	☐ Clinical data
☒	☐ Dual use research of concern

Methods

n/a	Involved in the study
☒	☐ ChIP-seq
☒	☐ Flow cytometry
☒	☐ MRI-based neuroimaging

Human research participants

Policy information about studies involving human research participants

Population characteristics	Ordered and resulted laboratory tests completed within the index date ± 2 years for Sutter Health Heart Failure (HF) or index date ± 3 years for "Geisinger NeuroScience Ischemic Stroke (GNSIS)" were used for imputation, where the index date was defined as the first time the disease of interest (i.e. ischemic stroke or heart failure) meet the diagnosis criteria28-31. Only quantitative laboratory values were considered for imputation.
Recruitment	The GNSIS database is composed of EHR data for patients with well-defined ischemic stroke from September 2003 to May 2019. The ICD-9-CM/ICD-10-CM diagnostic criteria for phenotypes was previously published. The comorbidity information based on ICD-9-CM or ICD-10-CM diagnosis was extracted within index data ± 3 years. Comorbidity was defined as a qualified diagnosis associated with either two outpatient visits or one inpatient visit. The entire laboratory data, based on Logical Observation Identifiers Names and Codes (LOINC), for the cohort were extracted and included in this study.<br>The Sutter Health HF database includes incidence heart failure cases identified from Sutter Health primary care population.31 Longitudinal EHR data were extracted on incidence cases diagnosed between January 1, 2010 to December 31, 2017. Encounter-based laboratory results with the corresponding LOINC identifiers within a two-year window before or after the index date were extracted. For the diagnosis domain, ICD-9 codes from outpatient office visits or phone visits were grouped using Clinical Classifications Software (CCS) [https://www.hcup-us.ahrq.gov/toolssoftware/ccs/ccs.jsp]. The CCS level 3 was adopted to group 5,379 ICD-9 codes into 363 unique CCS groups.

Ethics oversight	This study was approved by both Geisinger and Sutter Health Institutional Review Board.

Note that full information on the approval of the study protocol must also be provided in the manuscript.
